# Effect on Physical Activity of a Randomized Afterschool Intervention for Inner City Children in 3rd to 5th Grade

**DOI:** 10.1371/journal.pone.0141584

**Published:** 2015-10-28

**Authors:** Scott E. Crouter, Sarah D. de Ferranti, Jessica Whiteley, Sarah K. Steltz, Stavroula K. Osganian, Henry A. Feldman, Laura L. Hayman

**Affiliations:** 1 Department of Kinesiology, Recreation, and Sport Studies, The University of Tennessee, Knoxville, Tennessee, United States of America; 2 Department of Cardiology, Division of Outpatient Cardiology, Boston Children's Hospital, Boston, Massachusetts, United States of America; 3 Department of Exercise and Health Sciences, University of Massachusetts, Boston, Massachusetts, United States of America; 4 New Balance Foundation Obesity Prevention Center, Boston Children’s Hospital, Boston, Massachusetts, United States of America; 5 The Clinical Research Center, Boston Children’s Hospital, Boston, Massachusetts, United States of America; 6 College of Nursing and Health Sciences, University of Massachusetts, Boston, Massachusetts, United States of America; Vanderbilt University, UNITED STATES

## Abstract

**Background:**

Less than 45% of U.S. children meet the 60 min^.^d^-1^ physical activity (PA) guideline. Structured after-school PA programing is one approach to help increase activity levels. This study aimed to evaluate the feasibility and short-term impact of a supervised after-school PA and nutrition education program on activity levels.

**Methods:**

Forty-two 3^rd^-5^th^ graders from an inner-city school in Boston, MA were randomly assigned to a 10-wk after-school program of either: 1) weekly nutrition education, or 2) weekly nutrition education plus supervised PA 3 d^.^wk^-1^ at a community-based center. At baseline and follow-up, PA was measured using accelerometry and fitness (VO_2max_) was estimated using the PACER 15-m shuttle run. Additional measures obtained were non-fasting finger stick total cholesterol (TC) and glucose levels, waist circumference (WC), body mass index (BMI), percent body fat (%BF), and blood pressure (BP). Values are presented as mean±SE, unless noted otherwise.

**Results:**

Thirty-six participants completed the study (mean±SD; age 9.7±0.9 years). Participants attended >80% of the sessions. After adjusting for accelerometer wear time and other design factors, light and moderate-to-vigorous PA (MVPA) increased in the nutrition+PA group (+21.5±14.5 and +8.6±8.0 min^.^d^-1^, respectively) and decreased in the nutrition only group (-35.2±16.3 and -16.0±9.0 min^.^d^-1^, respectively); mean difference between groups of 56.8±21.7 min^.^d^-1^ (light PA, p = 0.01) and 24.5±12.0 min^.^d^-1^ (MVPA, p = 0.04). Time spent in sedentary behaviors declined in the nutrition+PA group (-14.8±20.7 min^.^d^-1^) and increased in the nutrition only group (+55.4±23.2 min^.^d^-1^); mean difference between groups of -70.2±30.9 min^.^d^-1^ (p = 0.02). Neither group showed changes in TC, BP, WC, %BF, BMI percentile, or fitness (p>0.05).

**Conclusions:**

The supervised afterschool community-based nutrition and PA program was well accepted and had high attendance. The changes in light PA and MVPA has potential to promote weight maintenance in inner-city elementary school children, however longer term studies with larger samples are needed.

**Trial Registration:**

ClinicalTrials.gov NCT01104038

## Introduction

Physical activity (PA) plays an important role in energy balance and obesity prevention [1]. However, U.S. national data indicate that only 42% of 6–11 year old children meet the current national guidelines for participating in at least 60 min^.^d^-1^ of moderate-to-vigorous physical activity (MVPA) [2]. Furthermore, there is a dramatic decrease in activity levels with age as only 8% of 12–15 year old adolescents obtain the recommended 60 min^.^d^-1^ of MVPA [2]. There are also gender and racial differences in PA levels with racial/ethnic minorities and girls having the lowest PA levels [3, 4].This was further emphasized in the 2014 U.S. National Report Card on PA for Children and Youth which gave a grade of D- for overall PA [5]. Meeting the PA guideline has been associated with improved cardiometabolic risk profiles [6, 7], glucose metabolism [8], and mental health [9] in children and adolescents; however, increased levels of light PA have also been shown to result in favorable changes in cardiometabolic biomarkers [7]. Given the low levels of those meeting the PA recommendation, particularly in health disparity children and adolescents, it is critical that there are strategies in place to increase opportunities for PA in youth. Supervised PA programs are often recommended as a way to improve cardiovascular fitness, increase energy expenditure, and aid in preventing weight gain for at risk youth [10–12].

The school setting has been a primary focus of numerous research investigations for increasing PA among youth via supervised exercise. In general, these school based programs have been shown to increase PA during physical education classes and outside of school, as well as improve fitness [13, 14]. However, the school setting faces challenges such as time constraints due to increased emphasis on standardized testing in reading and math. As a result, opportunities for PA during the school day have decreased [15]. Researchers working in urban settings and meeting with school officials may learn there are additional barriers to PA in urban schools including inadequate or nonexistent facilities such as no gymnasiums, tracks, or fields for exercise, as well as, potentially unsafe neighborhoods surrounding the schools [16–18].

To overcome time constraints and other environmental challenges inherent to some urban school-based PA programs, other programs, such as after-school programs, offered in safe community-based centers are needed for urban youth. In the US, approximately 8 million children attend supervised after-school programs that are conducted in the school or other community-based settings [19]. The past decade has witnessed increased attention to the importance of after-school programs in promoting PA in children and adolescents. Accumulated evidence supports the promise and potential of after-school programs in promoting PA and other health enhancing behaviors in children and adolescents [20, 21]. Minimal data exist, however, on the optimal after-school setting(s), the specific program intervention elements that work with younger versus older school age children, and the utility of such programs in promoting PA outside the after-school program setting [20, 22].

Particularly lacking is research on the feasibility of using community-based centers for conducting PA programs, especially in younger age groups [20]. Most programs have been conducted with older age groups (i.e., >5^th^ grade) and are located at a school and not an off-site community setting [13, 14]. Afterschool community-based PA programs could address urban barriers, raised by parents, such as cost, distance, and safety [16] as well as overcome previous limitations (e.g. parent transportation). While issues such as cost and transportation are still a burden to the after-school program, it helps to remove barriers to participation increasing the potential for more children and adolescents to become involved. One approach for how best to promote PA for elementary school-aged children in an underserved, urban setting, is to conduct community-based participatory research (CBPR). CBPR is a collaborative approach to research that involves the key stakeholders from the community in the research planning process [23]. The urban setting for PA promotion is an area that lends itself well to CBPR so that the researchers can collaborate with the key community stakeholders to determine how best to increase PA in their youth. A few studies have successfully utilized CBPR for PA promotion or obesity prevention in youth [24–27].

The purpose of this randomized controlled pilot study was to evaluate the feasibility and short term impact of a supervised after-school PA program for elementary school students. The primary objective was to increase PA outside of program time with secondary objectives of increasing fitness, decreasing BMI, and improving other CVD risk factors (i.e., blood pressure, total cholesterol, blood glucose). The program utilized a hybrid approach that incorporated programing that took place at both the school and a community-based center.

## Materials and Methods

### Development of Pilot Study

In preparation for this study, the researchers met with the school officials to determine how best to create and promote a PA program. The stakeholders included the school principal, physical education and health teachers, and the school-based family-community coordinator, a professional liaison to parents and community leaders. Consistent with the CBPR approach, results of individual and group meetings with these stakeholders guided and informed the design and implementation of the program. Specifically, they recommended that the program be held immediately after school at a community-based fitness facility and that transportation be provided to and from the fitness facility so that the parents could pick the children up at the school. With awareness of the increased prevalence in childhood obesity and concerns and observations about children’s dietary patterns, the stakeholders also requested that nutrition information was included in the program.

### Recruitment and Eligibility

The protocol for this clinical trial and supporting CONSORT checklist are available as supporting information; see S1 CONSORT Checklist and S1 Protocol. The protocol was registered at ClinicalTrials.gov (NCT01104038) On April 13^th^, 2010. Trial registration occurred after recruitment began in March 2010 due to oversight by the investigators, but once alerted to this issue action was taken immediately to register the trial. The authors confirm that all ongoing and related trials for this intervention are registered.

Participants were recruited from a single inner-city elementary school in the Boston Public Schools (BPS). English and Spanish recruitment materials, including informed consent and assent documents and a form collecting health information, socioeconomic status and contact information, were sent home from school. In addition, a study booth at the school’s open house was used to increase awareness of the study. The study was approved by the University of Massachusetts (UMass) Boston (December 3, 2009), Boston Children’s Hospital (December 7, 2009) and BPS (January 13, 2010) Institutional Review Boards. All parents gave written informed consent, and children provided written assent prior to participating in the study. The consent forms sent home with the recruitment materials were signed by the parents and returned to the study investigators and assent was obtained during the child’s first visit; after the study investigators discussed the study with the children and answered all questions.

Children were eligible for this study if they met the following inclusion criteria: 1) enrolled in the 3^rd^ through 5^th^ grade classrooms in the 2010 spring and fall semesters, 2) were English speaking, and 3) were able to attend after-school intervention sessions. Children were not able to participate in this study if the met one or more of the following exclusion criteria: 1) had medical conditions that would prohibit exercise, as indicated by lack of permission to participate in school physical education or by parent report, 2) if they had plans to move out of the area or change schools, or 3) if significant medical issues were identified during the initial study visit. Clearance from the child’s primary care provider was solicited by study staff whenever necessary. The protocol as designed called for 25 participants per group, providing 80% power to detect an effect size (difference ÷ SD) of 0.80, considered appropriate for an exploratory study.

### Measurements

Assessments were conducted on the UMass Boston campus at GoKids Boston, an interdisciplinary research, training and community-outreach facility designed to promote healthy lifestyle behaviors in children and youth, and at the elementary school under the supervision of trained staff and study investigators. All assessments were completed at baseline and follow-up (10 weeks) by study personnel. At baseline, all personnel conducting assessments were blinded to the intervention assignment; however, only 35% of the personnel were blinded at post-test assessments. Due to space limitations in the GoKids facility, the intervention was conducted in two waves; spring 2010 (recruitment and baseline testing: March; follow-up testing: June) and fall of 2010 (recruitment and baseline testing: September; follow-up testing: December). Seventeen participants were in the fall wave (9 nutrition+PA, 8 nutrition only) and 19 participants were in the spring wave (8 nutrition+PA, 11 nutrition only). Additionally, due to the number of children in each wave, the participants in each wave were split up into smaller groups so that each child could complete all measurements in a single afternoon.

#### Accelerometry

PA was assessed via a uniaxial accelerometer (model GT1M, ActiGraph, Pensacola, FL). The GT1M was initialized using 5-s epochs and to start collecting data on the afternoon that the participant received the device during the baseline or follow-up testing. Participants were asked to wear the GT1M for eight consecutive days during all waking hours except during water activities. The first day of measurement was not used due to it being a partial day, thus the maximum possible days of wear was seven days. The GT1M was attached to a nylon belt with the device positioned on the right hip and participants were provided with written and oral instructions on how to wear the belt and given a sheet for recording time when the device was worn.

After the accelerometer data were collected, they were cleaned and valid days were defined based on previously published criteria, using 60-sec epochs [2]. A valid day was defined as greater than 10-hrs of wear time with non-wear time defined as 60-min or more of consecutive zeros. To be included in the analysis participants needed to have a minimum of one valid measurement day at each time point. This was done to maximize the number of participants included in the analysis as requiring more days decreased the number of valid participants by greater than 25%. The wear time logs were used to verify the wear time for each day. Time spent per day in sedentary behaviors (<1.5 METs; <100 counts^.^min^-1^), light PA (1.5–2.99 METs) and MVPA (≥ 3.00 METs) were estimated using the Freedson age-specific child regression equation [28]. For the majority of participants (n = 28, 78%) the baseline and follow-up accelerometer assessment was performed during weeks when the participants were not attending the after-school program. Thus, for the participants that did wear the accelerometers while still attending the program (n = 8), the time spent engaged in the program was excluded from the analysis.

#### PACER

Cardiorespiratory fitness was assessed using the PACER 15-m shuttle run [29]. Participants ran between two sets of cones 15-m apart, as instructed by pre-recorded instructions set to music; beeps indicated when the participant should be at the next cone and the pace for each lap became progressively faster. If the participant failed to reach a cone in time, they were encouraged to speed up to get back on track with the test; the test was stopped when the participant failed to reach the cone for a second time. The total number of laps completed was used to estimate VO_2max_ using the equation of Léger et al [30].

#### Anthropometric and Laboratory Measures

Blood pressure (BP), height, weight, waist circumference (WC), and body composition were obtained according to standardized protocols by trained study staff [31, 32]. Following five minutes of quiet sitting, BP was measured by auscultation in the right arm three times, separated by one minute. The three BP measurements were averaged for analysis. Height and weight were measured (in light clothing without shoes) using a stadiometer and electronic scale (Tanita BC-418, Tokyo, Japan), respectively. Each measurement was taken twice and the average of the two values was used for the analysis. If the two measures for height or weight differed by more than 0.1 cm or 0.1 kg, respectively, then a third measurement was obtained and all three were averaged. Body mass index (BMI; kg/m^2^) was calculated and converted to gender- and age-specific BMI percentiles using the 2000 CDC growth charts [33]. Two measurements of WC were obtained (nearest 0.1 cm, average used for analysis) using a Gullick tape measure in a horizontal line between the supra-iliac crests at the end of a full expiration by the participant. Body composition was measured using bioelectrical impedance (Tanita BC-418, Tokyo, Japan), which has been shown to be a valid measure in children [34]. Non-fasting total cholesterol (TC) and glucose were measured by Cholestech LDX point-of-care finger stick procedures (Cholestech Corporation, Hayward, CA). Finger sticks were administered by a trained nurse and nursing student.

#### Self-Report Behavioral Measures

Dietary intake and PA questions, modified from the Youth Risk Behavior Survey (YRBS) [35] were used to assess usual intake of specific food categories, screen time and PA levels. Although the YRBS has not been validated in younger children, it was chosen for this study because it most closely assessed the behaviors relevant to the study.

### Intervention

After baseline data were collected, children were randomly assigned to either a nutrition only group or nutrition+PA group (intervention group) using a computer generated list provided by Boston Children’s Hospital Clinical Research Center. We employed permuted blocks and stratification by weight class to ensure balanced allocation over time between groups. To limit the attention bias, all children received some type of intervention (i.e. nutrition). In addition, since only one school was used for recruitment, the use of a true control group would increase the risk of contamination.

The nutrition only group received a 30-min weekly group nutrition education session for 10-wk, supported by printed dietary and motivational advice. The nutrition education sessions were held at the participant’s school on 10 consecutive Tuesdays. The sessions consisted of 15-min of education followed by 15-min of interactive activities that were based on the workshop topic for the week. The Food Guide Pyramid provided the workshop topics for the first 6-wks (grains, vegetables, fruit, dairy, meats and beans, and oils/fats). Other topics included beverages and snacks, dining out, mindful eating, and goal setting (see S1 Text). Workshop topics and interactive educational methods were adapted from Planet Health [36]. In addition, a healthy snack was provided each week that was selected from the food group discussed for that week. The sessions were taught by Boston Children's Hospital nutrition staff trained to deliver standardized healthy diet messages.

The intervention condition (nutrition+PA) received the nutrition education as described above in addition to an off-site PA program at GoKids Boston. The nutrition education was given on the same day for the nutrition only and nutrition+PA groups however, when one group was receiving the nutrition education the other group was in a different classroom working on homework. The GoKids PA program used behavioral strategies based in social-cognitive theory (SCT) [37] and provided supervised PA for children. The program included an orientation to GoKids and all of the equipment, consistent staff encouragement and reinforcement, and an SCT take-home booklet at the end of the intervention designed to help children continue PA beyond the intervention. Students were transported by bus from their school to GoKids for the PA program and back to their school at the end of each day; school staff remained with the children until picked up by a caretaker. PA sessions were 60-min in duration and occurred three afternoons per week for 10-wk. Since recruitment was done in two waves, there were no more than nine participants attending the PA sessions at a given time. Sessions included a variety of PA using treadmills, elliptical machines, strength training equipment, and interactive video games that were supervised by trained GoKids staff. All participants completed a 10-min warm-up using the SportWall for a group activity. To facilitate continuous engagement in activity during the program, participants were split into groups of 3–4 and completed a circuit of the following activities, with each group starting at a different station: 1) Treadmill, ArcTrainer, or Bike up to 15-min total, 2) Weights: 8 to 20 reps per machine, 5 machines, in 1 or 2 rounds as appropriate for the child, and 3) 10-min of exergaming. Time permitting, participants were allowed ‘free time’ to play any additional activity of their choosing (e.g., jump rope, dodgeball, interactive games such as Trazer, Jackie Chan, Dance Dance Revolution, Wii, treadmill walking, etc).

#### Process and Feasibility Measures

Attendance was collected at each exercise and nutrition education session. At exercise sessions, participation duration was captured for each activity for each child. Children used a 9-point facial affective scale to rate specific activities in which they participated, at the conclusion of each exercise session [38]. At the end of the intervention, all participants were surveyed regarding how well they liked or did not like the program and were asked open-ended questions about what had been their favorite and least favorite aspects of the program. The school principal also provided informal feedback about the program.

### Data Analysis

We compared baseline characteristics of the two groups by Fisher’s exact test. For anthropometrics and laboratory measures, we tested for significant mean change within each group using the one-sample t-test and compared mean change between groups by the independent t-test. We adjusted the between-group comparison for age and tested for effect modification by wave (fall or spring) and weight stratum (above or below BMI 85th percentile) using factorial analysis of variance. Significance of the comparison was unaffected by adjustment for age or effect modification by wave or weight stratum.

The wave was not a significant effect modifier for light PA (p = 0.70) or sedentary behaviors (p = 0.29). For MVPA, wave was a significant effect modifier (interaction p = 0.03) and wave-specific results are reported when necessary. We analyzed PA and fitness data by factorial analysis of variance, with group, time, and group × time interaction as primary factors, adjusting additionally for weight stratum, enrollment wave, effect modification as needed, and in the case of accelerometry, day of the week (weekday vs. weekend) and daily wear-time. From parameters of the fitted model we constructed adjusted estimates of baseline mean, follow-up mean, and mean change for each group, as well as the net change (mean change in nutrition+PA participants minus mean change in nutrition only participants), representing group × time interaction, our primary hypothesis. Results with two-sided p<0.05 were considered statistically significant. SAS software (version 9.2) was used for all computations.

## Results

### Participant Characteristics

**Fig 1:**
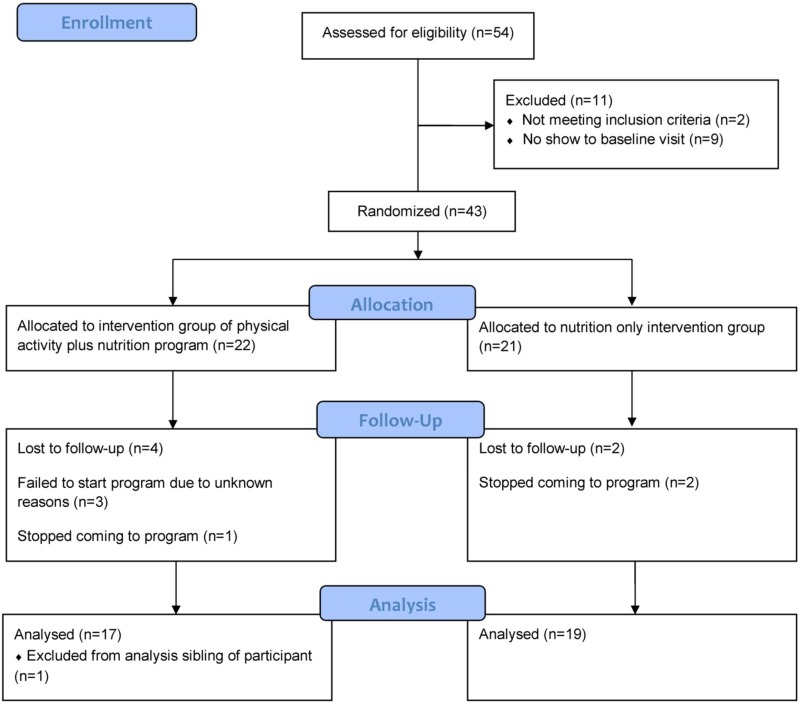
Consort diagram.

Recruitment materials for the study were distributed to a total of 342 families that had students in the eligible classrooms during the study periods; 54 (15.8%) responded and were assessed for eligibility in the study. Forty-three children were randomized into the study and 36 completed the study (Fig 1). Nine participants (17%) declined to continue prior to being randomized in the study and after randomization, four participants in the nutrition+PA group and two participants in the nutrition only group dropped out of the study. A sibling of one of the participants was allowed to participate in the PA program for the convenience of the family, but was excluded from the analysis to ensure independence of observations. There were no differences in the demographics and anthropometrics between those that completed the study and those that dropped out. The complete de-identified data set used for analysis is available as S1 File.

At baseline, there were no differences between the nutrition+PA and nutrition only group for age (9.6 yrs vs 10.0 yrs, respectively) and each group had nine normal weight (42.8%; BMI<85^th^ percentile), five overweight (23.8%; BMI ≥ 85^th^ to < 95^th^ percentile), and seven obese (33.3%; BMI ≥ 95^th^ percentile) participants. Tables 1 and 2 show the socio-demographic and anthropometric characteristics, respectively, for those with a baseline and follow-up measurement. 53% of the participants were male, 70% were from minority racial/ethnic backgrounds, 80% of the participants received free and reduced price school lunch, and 60% were from single parent households. Non-fasting laboratory parameters were generally in the normal range for age and did not differ between conditions.

**Table 1:** Sociodemographic characteristics for all enrolled participants with baseline and follow-up measurements (n = 36).

		Nutrition Only (n = 19)		Nutrition+PA (n = 17)	
		n	%	n	%
**Gender**	Male	9	47.4	10	58.8
**Grade**	Third	5	26.3	10	58.8
	Fourth	9	47.4	5	29.4
	Fifth	5	26.3	2	11.8
**Race**	White/Caucasian	6	31.6	5	29.4
	Black/African-American	9	47.4	9	52.9
	Asian	2	10.5	1	5.9
	Unknown*	2	10.5	2	11.8
**Ethnicity**	Hispanic	9	47.4	11	64.7
**Family composition**	Two parents	4	21.1	7	41.2
	Single parent	13	68.4	9	52.9
	Grandparents	2	10.5	0	0.0
	Other	0	0.0	1	5.9
**Medical conditions**	Asthma	4	21.1	3	17.7
	Allergies	4	21.1	2	11.8
**Medical care**	Doctor’s office	3	15.8	0	0.0
	Hospital	2	10.5	4	23.5
	Community health center	14	73.7	13	76.5
**Parental employment**	Employed (≥35 hours)	6	31.6	6	35.3
	Employed (<35 hours)	1	5.3	3	17.6
	Student/not employed	10	52.6	8	47.1
	Other/missing	2	10.5	0	0.0
**Parental education**	Some high school	4	21.1	4	23.5
	High school/GED	5	26.3	5	29.4
	Some college	4	21.1	2	11.8
	2-year college degree	2	10.5	0	0.0
	4-year college degree	3	15.8	1	5.9
	Other/missing	1	5.2	5	29.4
**Free/reduced school lunch**		14	74	15	88

PA, physical activity.

*Self-report, no race designation provided.

**Table 2:** Anthropometric characteristics and non-fasting lab values for baseline and follow-up time periods by group assignment (Mean ± SD), and change in parameters (Mean ± SE).

Characteristic	Group	Baseline	Follow-up	Change*	p†
**Height (cm)**	Nutrition+PA	135.5 ± 9.2	136.7 ± 9.2	1.2 ± 0.1	0.11
	Nutrition Only	138.9 ± 11.5	140.4 ± 11.5	1.5 ± 0.2	
**Weight (kg)**	Nutrition+PA	40.4 ± 15.6	41.2 ± 16.0	0.7 ± 0.3	0.54
	Nutrition Only	40.8 ± 12.6	41.3 ± 12.6	0.5 ± 0.3	
**Waist circumference (cm)**	Nutrition+PA	69.4 ± 13.9	68.9 ± 14.3	–0.5 ± 0.7	0.87
	Nutrition Only	68.0 ± 10.9	67.4 ± 10.2	–0.7 ± 0.5	
**BMI (kg** ^**.**^ **m** ^**-2**^ **)**	Nutrition+PA	21.7 ± 7.3	21.8 ± 7.4	0.0 ± 0.2	0.36
	Nutrition Only	20.7 ± 3.6	20.5 ± 3.6	–0.2 ± 0.2	
**BMI percentile**	Nutrition+PA	77.7 ± 24.5	75.5 ± 25.4	-2.2 ± 1.2	0.67
	Nutrition Only	77.5 ± 27.4	75.9 ± 27.0	–1.6 ± 0.2	
**Body fat (%)** ‡	Nutrition+PA	26.9 ± 7.9	27.0 ± 8.1	0.1 ± 0.4	0.53
	Nutrition Only	29.5 ± 5.9	29.2 ± 6.3	–0.3 ± 0.4	
**Systolic BP (mmHg)** ‡	Nutrition+PA	95 ± 15	94 ± 15	–1 ± 2	0.32
	Nutrition Only	96 ± 9	98 ± 12	2 ± 2	
**Diastolic BP (mmHg)** ‡	Nutrition+PA	58 ± 8	58 ± 8	0 ± 2	0.80
	Nutrition Only	60 ± 6	61 ± 10	1 ± 2	
**Total cholesterol (mmol** ^**.**^ **L** ^**-1**^ **)** ‡	Nutrition+PA	4.3 ± 0.4	4.4 ± 0.6	0.1 ± 0.2	0.83
	Nutrition Only	4.1 ± 0.5	4.2 ± 0.6	0.1 ± 0.2	
**Glucose (mmol** ^**.**^ **L** ^**-1**^ **)** ‡	Nutrition+PA	4.6 ± 0.5	5.1 ± 0.8	0.6 ± 0.2	0.36
	Nutrition Only	4.7 ± 0.6	5.0 ± 0.8	0.3 ± 0.2	

* Unadjusted mean change ± standard error. All within-group mean changes were non-significant, p>0.05.

^†^ From Student t-test comparing mean change in nutrition+PA and nutrition only participants (N = 36). Significance of the comparison was unaffected by adjustment for age or effect modification by wave or weight stratum.

^‡^ For change in percent body fat by bioelectrical impedence testing: nutrition only n = 16; nutrition+PA n = 16. For blood pressure: nutrition only n = 18; nutrition+PA n = 17. For labs: nutrition only n = 16; nutrition+PA n = 15.

BMI, body mass index; BP, blood pressure.

### Physical Activity and Fitness

Thirty-one participants (15 nutrition+PA group and 16 nutrition only group) had a minimum of one valid measurement day at each time point and were included in the accelerometer analysis. Requiring a minimum of two or three valid days would have decreased the sample size to 23 and 20 participants, respectively. After excluding invalid days, the accelerometer was worn, on average, for (mean ± SD) 4.9 ± 1.9 days during the baseline measurement period, and 3.6 ± 2.1 days in the final measurement period. Children in the nutrition+PA group wore the accelerometers for approximately the same number of minutes per day at both measurement times, while the nutrition only group wore the accelerometers on average 59 min^.^d^-1^ less at the follow-up measurement (interaction p = 0.01).

**Fig 2:**
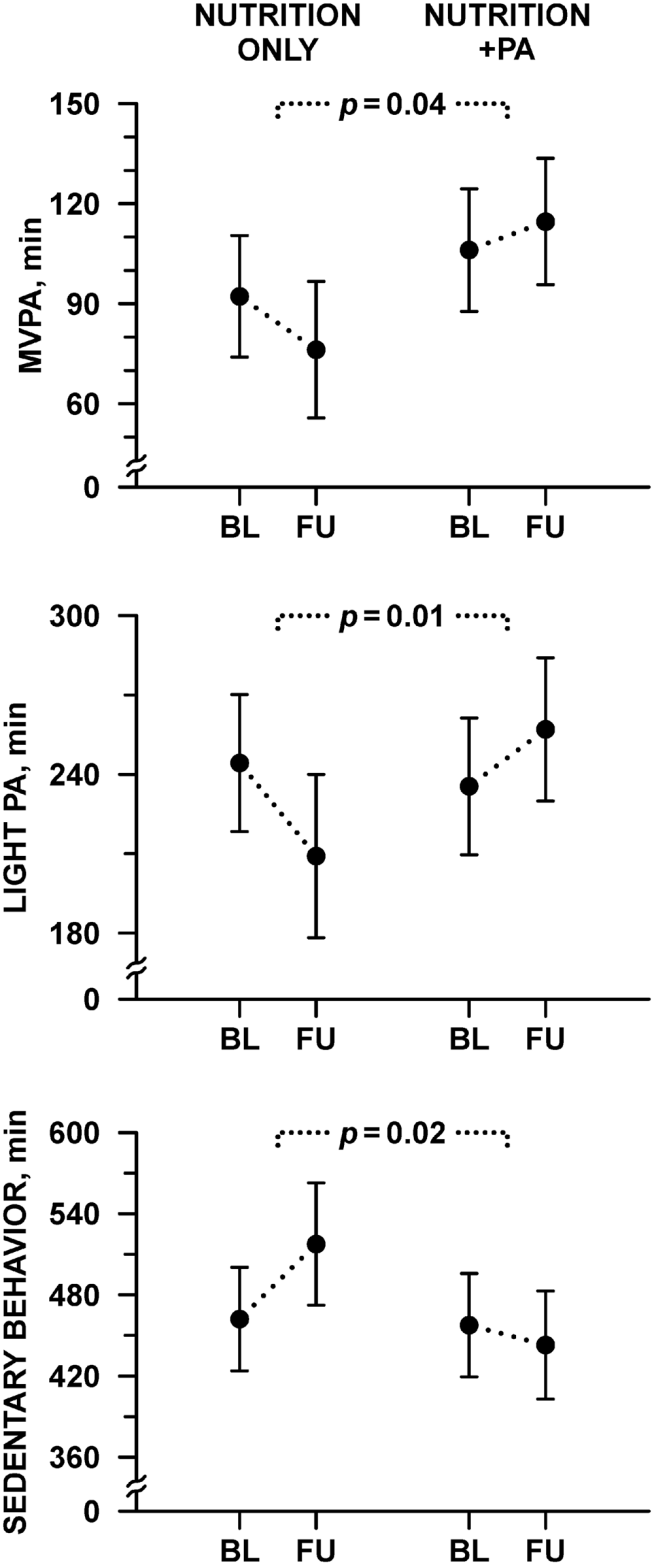
Physical activity and sedentary behavior as measured by accelerometry. Adjusted means with 95% confidence interval at baseline (BL) and follow-up (FU), compared between nutrition+PA and nutrition only. Vertical axes with suppressed zero are employed to highlight changes.

Time spent in sedentary behaviors, light PA and MVPA are shown in Fig 2 and Table 3 by group assignment and time point. There were no differences between groups at baseline for sedentary time (p = 0.86), light PA (p = 0.61), and MVPA (p = 0.27). MVPA increased in the nutrition+PA group by 8.6 ± 8.0 min^.^d^-1^ (mean change ± SE; p = 0.29) and decreased in the nutrition only group by 16.0 ± 9.0 min^.^d^-1^ (p = 0.08) and the difference between the groups was significant (24.5 ± 12.0 min^.^d^-1^, p = 0.04). The effect on MVPA was significantly modified by wave (fall or spring; interaction p = 0.03). Wave-specific mean differences were 51.8 ± 17.3 min^.^d^-1^ for the spring wave (nutrition only group: -16.7 ± 16.0 min^.^d^-1^; nutrition+PA group: +35.0 ± 11.4 min^.^d^-1^) and -0.3 ± 16.0 min^.^d^-1^ for the fall wave (nutrition only group: -15.2 ± 11.9 min^.^d^-1^; nutrition+PA group: -15.5 ± 10.9 min^.^d^-1^). Light PA increased in the nutrition+PA group (+21.5 ± 14.5 min^.^d^-1^) and decreased in the nutrition only group (-35.2 ± 16.3 min^.^d^-1^), and the difference between the groups was significant (56.8 ± 21.7 min^.^d^-1^, p = 0.01). Time spent in sedentary behaviors declined in the nutrition+PA group (-14.8 ± 20.7 min^.^d^-1^) and increased in the nutrition only group (+55.4 ± 23.2 min^.^d^-1^), and similarly, the difference between the groups was significant (-70.2 ± 30.9 min^.^d^-1^, p = 0.02).

**Table 3:** Accelerometry and fitness data. Unadjusted mean ± SD at baseline and follow-up by group assignment; mean change within group and difference between groups ± SE, adjusted for wear time and design parameters.

Characteristic	Group	Baseline	Follow-up	Change*	p†
**MVPA (min** ^**.**^ **d** ^**-1**^ **)**	Nutrition+PA	101.6 ± 70.0	114.1 ± 59.7	8.6 ± 8.0	0.29
	Nutrition Only	86.1 ± 48.9	72.1 ± 35.3	–16.0 ± 9.0	0.08
	Difference	—	—	24.5 ± 12.0	0.04
**Light PA (min** ^**.**^ **d** ^**-1**^ **)**	Nutrition+PA	236.7 ± 86.1	261.3 ± 72.5	21.5 ± 14.5	0.14
	Nutrition Only	248.3 ± 91.3	212.0 ± 103.0	–35.2 ± 16.3	0.03
	Difference	—	—	56.8 ± 21.7	0.01
**Sedentary behavior (min** ^**.**^ **d** ^**-1**^ **)**	Nutrition+PA	466.6 ± 155.4	441.2 ± 154.0	–14.8 ± 20.7	0.48
	Nutrition Only	486.5 ± 155.7	479.2 ± 158.0	55.4 ± 23.2	0.02
	Difference	—	—	–70.2 ± 30.9	0.02
**Estimated VO**_**2max**_ **(ml** ^**.**^ **kg** ^**-1.**^ **min** ^**-1**^ **)**	Nutrition+PA	42 ± 3	41 ± 3	–0.5 ± 1	0.34
	Nutrition Only	40 ± 2	40 ± 3	–0.5 ± 1	0.41
	Difference	—	—	–0.1 ±0.8	0.92

* Change estimates from mixed-model analysis of variance, adjusted for wear time, weekday vs. weekend, normal weight vs. overweight, and wave (spring or fall).

^†^ In nutrition+PA and nutrition only rows, p tests for non-zero change within group. In Difference row, p tests whether the two groups changed differently.

PA, physical activity; MVPA, moderate-to-vigorous PA.

**Fig 3:**
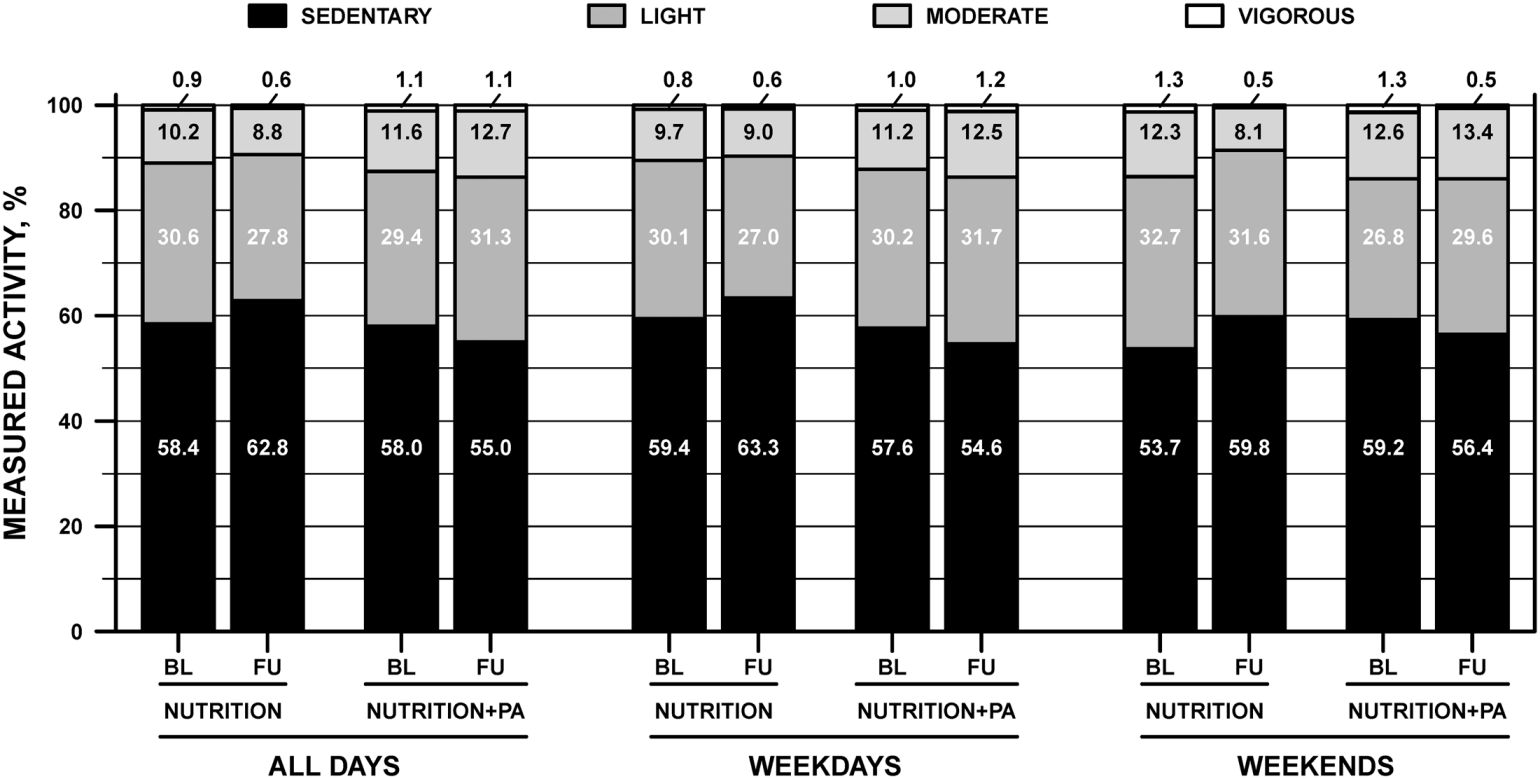
Proportion of time spent in sedentary behaviors and light, moderate, and vigorous physical activity (PA), as measured by accelerometry, at baseline (BL) and follow-up (FU) for the nutrition and nutrition+PA groups across all days, weekdays, and weekend days.

There were no differences between weekdays and weekend days for time spent in MVPA, light PA or sedentary behaviors (Fig 3; p = 0.42, p = 0.59, and p = 0.84, respectively). When the participants were categorized by weight status, the normal weight children participated in 37 ± 12 min^.^d^-1^ more MVPA compared to overweight children (116 ± 9 vs. 79 ± 8 min^.^d^-1^, respectively; p = 0.005). For time in sedentary behaviors, the normal weight children spent 52 ± 23 min^.^d^-1^ less compared to overweight children (444 ± 19 vs. 496 ± 16 min^.^d^-1^, respectively; p = 0.04). There was no difference between the normal weight and overweight children for time spent in light PA per day (244 ± 13 vs. 229 ± 11 min^.^d^-1^, respectively; p = 0.36). For fitness, there were no significant within- or between-group differences in estimated VO_2max_ (p>0.30) (Table 3).

### Cardiovascular risk factors

In the sample as a whole, BMI percentile declined (mean±SE; -1.9 +- 0.7, p = 0.01); however, there was no significant difference in change in BMI percentile between groups (p>0.05). There were no significant changes baseline to end of study in TC, BP, WC, or %BF (p>0.05) (Table 2). Adjusting for baseline weight status, age and gender did not alter these results.

### Lifestyle behaviors

At baseline, less than half (42%) consumed the recommended servings of fruit per day, but 61% reported eating vegetables two or more times per day. Half of the participants reported eating with their families at least once per day (47%). One third of participants drank more than one serving of 100% fruit juice every day, and 23% drank soda two or more times per day. There were no significant changes in either group between baseline and the final visit for reported dietary quality, with the exception of fruit intake, which decreased in the nutrition+PA group (p = 0.043).

### Attendance and acceptability

Overall, attendance at both the PA and nutrition sessions was >80%. A majority of the children randomized to the nutrition+PA felt the frequency of sessions was just right (71%). Most (93%) rated the intervention as the highest score (9 out of 9), and reported the intervention made them want to exercise more (93%). The majority of children in the nutrition+PA and the nutrition only group reported the nutrition education sessions helped them improve their eating (86% and 100%, respectively). There were no adverse events.

## Discussion

A primary goal of this randomized controlled pilot study of urban elementary school children was to determine if children participating in a supervised community-based nutrition plus PA program had an increase in PA, outside of program time, compared to those who received a nutrition only program. Participants in the nutrition+PA program had positive changes in sedentary time, light PA and MVPA, compared to the nutrition only group. However, there were no changes in body composition, fitness, TC or BP during the 10-wk pilot intervention. This study demonstrated feasibility, good compliance and considerable enthusiasm on the part of the child participants.

The changes seen in sedentary behaviors, light PA and MVPA have health significance. The change in MVPA suggests that the nutrition+PA group obtained 25 min^.^d^-1^ more compared to the nutrition only group. This represents 41% of the recommended 60 min^.^d^-1^ and is a significant step in reaching the PA recommendation. This is in line with other studies that have shown children to accumulate large amounts of activity in after-school periods. Trost and colleagues showed that after-school programs provided approximately 20-min of MVPA daily, which is in line with the changes seen in this program [39]. Given children obtain MVPA through other activities such as recess and physical education, the addition of after-school PA programming has the ability to provide additional time to be active especially in urban youth who many times have decreased PA opportunities due to environmental barriers.

Prolonged periods of sedentary behaviors have been highlighted as an independent risk factor for obesity and other chronic health conditions [40], and replacing sedentary time with even light PA has potential to improve health outcomes [41–43]. A small daily energy imbalance may lead to substantial weight gain over time [44], and an intervention such as this one has potential to close the energy gap imbalance and prevent further weight gain. Our study participants were at greater risk than the general population due to high rates of overweight and obesity (23.8% and 33.3% respectively, 57.1% combined), compared to Massachusetts school children overall (44% of 4^th^ graders) [45]. This makes the changes in sedentary time, light PA and MVPA all the more important. Few effective evidence-based interventions to normalize weight status or maintain weight loss are currently available [46]. Attention has turned to “bending the curve” of already obese populations or better yet, preventing its emergence. Assuredly, a combined approach to PA and nutrition will be necessary [36, 47–49], and interventions such as this could be combined with other efforts in school and at home as a pathway for success.

One potentially important finding for the changes seen in PA are those of the nutrition only group. While the nutrition+PA group significantly increased their PA and decreased their sedentary time compared to the nutrition only group, these changes were primarily due to large and significant decreases in light PA and MVPA and an increase in sedentary time of the nutrition only group. This contrasts with the view that if no intervention is given, as in the case of the nutrition only group, it would be expected that the PA would stay the same. An important consideration in this is that the accelerometer measurement was intended to capture changes in PA outside of program time, thus it does not take into account the time spent in PA during the intervention. Therefore, the changes in PA seen underestimate the actual increase in PA of the nutrition+PA group during the course of the intervention.

Other factors to consider when examining the changes seen between the nutrition only and nutrition+PA groups are environmental factors and accelerometer wear time. One potential issue is how weather affects outdoor activity. This study recruited over two different waves; a fall wave which ended in December and a spring wave which ended in June. It is possible that the fall wave decreased their PA due to the colder weather in December. This is especially apparent in the analysis of time spent in MVPA per day were there was a wave effect. During the fall wave both groups decreased their MVPA by approximately 15 min^.^d^-1^. In contrast, during the spring wave the nutrition only group decreased their MVPA by 16.7 min^.^d^-1^ while the nutrition+PA group increased their MVPA by 35 min^.^d^-1^. This suggests that, unlike time spent in sedentary behaviors and light PA, the nutrition+PA group is the driving factor in the change seen for MVPA. It also appears different seasons that the follow-up measurements occurred in may be playing an important factor by potentially decreasing outdoor time in the winter months. This has important implications in that it may be more important to have structured PA programs during the winter months when the weather is colder and less day light, which may decrease motivation levels for children to find opportunities to be activity in their free-time.

Compliance with wearing the accelerometers may also be a contributing factor to the differences seen between groups. The nutrition only group did not wear the accelerometer the same amount of time at baseline and follow-up. Thus, when the analysis was controlled for wear time it had a larger effect on the nutrition only group compared to the nutrition+PA group which wore the accelerometer for the same amount of time at each time point. In addition, only a minimum of one valid accelerometer wear day was required at baseline and follow-up. This could further affect the outcomes of the study if the valid day used was not representative of their typical daily activity pattern. Lastly, in children it is known that PA declines with age. While the changes seen in the nutrition only group are not likely due to an aging effect, it does point to the notion that in children and adolescents it is even more important to intervene early. The changes seen in this pilot study suggest that if no intervention is given, PA will continue to decrease as children transition to adolescence. While the PA program did have an effect on increasing overall activity and decreasing sedentary time in the nutrition+PA group outside of the program time, it appears that other factors may be influencing this change. This suggests that more structured programming is needed to increase PA on a daily basis. In addition, more intensive PA education is needed as well as methods to promote changing PA behavior outside of program time.

Acceptability in PA programs is always a primary concern. Previous research has shown that one reason for low attendance at community-based programs is due to the burden placed on the parent for transporting their child to the program [50]. There is also evidence to suggest that greater improvement in outcome measures are seen in those that attend greater than 40% of PA programing sessions [20]. In this study, attendance in the program and our satisfaction measures indicated that the program was indeed acceptable to the participants. In the current study, we had greater than 80% attendance at both the PA and nutrition education sessions. This could potentially be attributed to two factors: 1) the nutrition programing was completed at the school requiring no additional transportation, and 2) for the PA program the participants were bussed from their school to the GoKids center and back to their school with school staff supervision. This allowed their parents to pick up their children at their school, at the end of a typical work-day without the additional burden of transportation to the GoKids center. Acceptability of the program was also high, with 93% of participants rating the intervention as 9 out of 9 on the satisfaction scale, suggesting that the program was well-liked, and indeed acceptable to the kids attending the program. Future studies should further examine ways that under resourced, inner-city schools might promote PA and nutrition while decreasing the burden on parents to increase attendance rates by utilizing the school setting in after-school hours when possible and providing transportation to participants.

There were several limitations to this feasibility study. It could be argued that our nutrition only group was not appropriate in that there was less attention paid to the nutrition-only cohort (e.g., no bus ride or fun new activity). Differences in the intervention could be attributed to this factor rather than the intervention itself. A second limitation, relates to the low compliance in wearing the accelerometers that resulted in only requiring a minimum of one valid day at baseline and follow-up. This most likely is not representative of their typical activity throughout the week and has potential to influence the PA changes seen in the study. For example if the baseline measurement was on a more active day then the follow-up measurement the decrease in PA may be overestimated. A third limitation relates to the unique setting in which the intervention took place. GoKids is an exercise facility designed for children and adolescents, incorporating pediatric sized equipment with a focus on active and engaged play. These types of facilities are not currently available in most communities. Lastly, we did not measure the intensity of activities being performed during the PA programming. Thus, it is not known if the activity intensity was sufficient to see changes in fitness and cardio-metabolic markers measured in the study.

Despite these limitations, the current study suggests several important lessons about conducting a community-based intervention to promote PA. First, attendance in a community-based after-school PA program is likely to be enhanced if transportation is provided. Feasibility of the program may also increase if stakeholders, such as school personnel, are involved and promote the activity, including the school nurse, community liaisons, and school principals. As the literature would suggest, incorporating interactive video games into the PA program may increase enjoyment and overall PA participation [51]. Although we were not able to demonstrate change in specific metrics related to attitudes and barriers toward PA, we suspect this was related to inadequate existing metrics for this age group as well as the relatively short duration of the intervention. Future studies need to determine if increasing intensity and/or volume of PA, increasing the amount of exergaming, or if including parents in the nutrition education and/or PA parts of the program can better impact fitness and risk profiles.

### Conclusion

The Gokids community-based after-school PA and nutrition program resulted in positive differences in sedentary time, light PA, and MVPA for those in the nutrition+PA group compared to those in the nutrition only group. The positive differences may have been attributed to maintaining physical activity levels in the nutrition+PA group while they were declining in the nutrition only group. As a pilot study, this program also was deemed to be acceptable as indicated by the high attendance and satisfaction ratings. BMI and other cardiovascular risk factors were not measurably affected in this small sample size; more prolonged and/or intensive exercise may be required to see further changes.

## Supporting Information

S1 CONSORT Checklist Consort Checklist. (DOC)

S1 File Deidentified data used for analysis in manuscript. (XLS)

S1 Protocol Trial Protocol. (PDF)

S1 Text Nutrition education lessons. (PDF)
