# Supplementary material for: Effect on Physical Activity of a Randomized Afterschool Intervention for Inner City Children in 3rd to 5th Grade
Source: PLoS One. 2015 Oct 28;10(10):e0141584. doi: 10.1371/journal.pone.0141584 (PMC4624808; doi:10.1371/journal.pone.0141584)
Supplement: S1 Protocol — (PDF) [file pone.0141584.s003.pdf]

Change in Cardiometabolic Risk Factors during an Interactive Fitness Program (EXCEL Study)

Manual of Operations

Table of Contents

I. Study Overview 2
- **a. Study Aims and Objectives** 2
- **b. Background and Rationale** 2
- **c. Timeline** 3

II. Study Organization 4
- **a. Study Collaborator Roles** 4
- **b. Contact Information** 4

III. Recruitment 5
- **a. Inclusion and Exclusion Criteria** 5
- **b. Recruitment Procedures** 5
- **c. Screening** 6
- **d. Informed Consent** 6
- **e. Baseline Assessment** 7
- **f. Incentives** 7

IV. Study Visits 7
- **a. Study Visit Schedule** 7
- **b. Intervention Protocol** 8
- **c. Randomization Procedures** 8
- **d. Study Close-out** 9

V. Study Measurements and Data Collection 9
- **a. Administering Surveys/Questionnaires** 9
- **b. Procedures and Examinations** 10
- **c. Sample Collection** 10
- **d. Case Report Form Completion** 11
- **e. Study Monitoring** 12

VI. Adverse Event Reporting 12
- **a. Risks and Discomforts** 12
- **b. Reporting and Follow-up Procedures** 12

VII. Data Management Procedures 13
- **a. Overview of Data Flow** 13
- **b. Data Entry Procedures** 13
- **c. Form Completion Tracking** 13
- **d. Data Entry Error Resolution and Editing Procedures** 13

VIII. Quality Control 13
- **a. Training and Certification** 13
- **b. Laboratory Quality Control** 13
- **c. Communication Procedures** 14

IX. References 14

I. Study Overview

a. Study Aims and Objectives

Primary Aim:
To determine the change from baseline in minutes per day spent in moderate or vigorous physical activity (MVPA) as measured by accelerometry. We hypothesize that the *GoKids* intervention will more effectively increase time spent in MVPA compared to baseline vs. the *Advice-Only* condition.

Secondary Aims:
Cardiorespiratory fitness will improve in the group receiving the *GoKids* intervention more than in the group in the *Advice-Only* condition.
Endpoint: Cardiorespiratory fitness will be assessed in both groups pre- and post intervention using a modified FitnessGram test. FitnessGram tests will be supervised by Dr. Crouter, who will also interpret these data.

Anthropometric characteristics will improve in the group receiving the *GoKids* intervention more than in the group receiving *Advice-Only*.
Endpoints: Percent body fat by BIA, degree of obesity by BMI percentile, waist circumference, blood pressure. Drs. De Ferranti, Osganian and Hayman will interpret the anthropometric data.

Non-fasting total cholesterol (TC) and HDL will improve from baseline in the group receiving the *GoKids* intervention more than in the group receiving *Advice-Only.*
Endpoint: Change in Cholestech TC and HDL from baseline. Dr. de Ferranti will evaluate and follow-up lipid parameters.

Psychosocial measures of physical activity and self-perception will be positively affected by participation in *GoKids* more than the *Advice-Only* condition, including physical activity self efficacy, physical activity social support, physical activity perceived barriers, physical activity enjoyment, competence and global self-worth.
Endpoints: Physical activity self efficacy, physical activity social support, physical activity perceived barriers, and physical activity enjoyment will be measured with scales that were validated for use in the TAAG study with 6th grade children (Dishman 2009). Competence and global self-worth will be measured with the modified Harter Self-Perception Profile Scale (Harter 1985). Dr. Jessica Whiteley will analyze and interpret these data.

Exer-gaming is a feasible way to increase moderate and vigorous physical activity in elementary aged children.
Endpoints: Successful recruitment of students, extent of implementation of intervention activities measured by student attendance/participation rates in intervention, student's level of enjoyment in or satisfaction with the intervention activities, retention rate in the study, costs to implement the intervention, barriers to and facilitators of implementation.

Sub-Study Aims (exploratory):
The *GoKids* intervention group will show greater improvement in CVD risk measurements, including vascular reactivity, fasting lipid and glucose metabolism parameters, resting energy expenditure, left ventricular hypertrophy, and inflammatory measures compared to the *Advice-Only* condition.
Primary Endpoint: change in insulin resistance as measured by HOMA-IR.

b. Background and Rationale

Obesity is increasingly common in childhood and adolescence, and is a major threat to health. Childhood obesity is predictive of risk for chronic conditions, including type 2 diabetes and cardiovascular disease (CVD) in adulthood (Morrison 2008), and obese pediatric patients have high rates of cardiovascular risk factors during childhood. Declining levels of physical fitness among children and adolescents contribute to the obesity-associated health risks of future generations (American Heart Association 2005). Physical activity is known to have physiologic benefits in many areas; recommendations to increase physical activity levels in children are evidence-based (Strong 2005).

Exercise training is a type of physical activity that can improve physical fitness. Exercise training improves a host of physiological risk factors and provides psychosocial benefits for children who are at increased CVD risk, including those with obesity (Klijn 2007) and diabetes (Caranti 2007). Furthermore, several recent pediatric studies suggest that exercise training reduces cardiometabolic risk factors (Ferguson 1999) and the intra-individual clustering of cardiometabolic risk factors (Reinehr 2009). Reduction in risk factors may occur both by direct effects (e.g. blood pressure reduction, increasing HDL), and indirectly through weight reduction. Despite evidence of its benefits, exercise training prescribed by physicians for overweight children is traditionally vague, developmentally inappropriate, and/or fraught with psychological financial & practical barriers. Recommendations may not be supported by sufficient motivational assistance.

Recently there has been increased interest in the use of interactive and new technologies to promote physical activity, prevent or reverse unhealthy weight, and improve physical fitness in youth (Whiteley 2007; An (in press); Daley 2009). These technologies include popular video games that feature player movement, physiologic monitoring devices (e.g., pedometers and heart rate monitors), and internet mediated counseling. Initial studies in adults demonstrate that interactive technologies can intensify antismoking attitudes, improve prevention behaviors, influence dietary habits, increase physical activity, strengthen adherence to medical treatment plans, or improve chronic disease self-management (Norman 2007). The few published pediatric studies of exer-gaming include design limitations such as: 1) small sample sizes; 2) inadequate measures of physical activity and body composition (Trost 2008), and 3) an absence of the use of evidence-based behavioral strategies, important components of interventions designed to increase and sustain physical activity (Hayman 2006). Published exercise studies have not included children at increased CVD risk. “Exer-gaming” techniques should be tested in pediatric patients with CVD risk factors to determine if they increase physical activity, and whether this increases physical fitness, decreases body fat, and reduces CVD risk factors.

c. Timeline

2010

Study Event	Jan	Feb	Mar	Apr	May	Jun	Jul	Aug	Sep	Oct	Nov	Dec
Recruitment		X				X			X			
Consent		X				X			X			
Screening		X				X			X			
Baseline			X						X			
Randomize			X						X			

Intervention and Advice-Only sessions			X	X	X				X	X	X	
12-week assessment						X						X

II. Study Organization

a. Study Collaborator Roles

Collaborators at Children's Hospital Boston

Role	Name	Title
Principal Investigator	Sarah de Ferranti, MD, MPH	Director, Preventive Cardiology
Co-Investigator	Stavroula Osganian, MD, ScD, MPH	Director, Clinical Research Program
Nurse	Annette Baker, NP	Preventive Cardiology Clinic Nurse Practitioner
Project Manager	Sarah Krathwohl, MPH	Clinical Research Specialist
Lead Recruiter	Tracy Antonelli, MPH	Clinical Research Study Manager
Clinical Research Coordinator	Mark Berry, MS	Clinical Research Coordinator
Data Manager/Data Entry	Moriah Polanco	Data Coordinator
Clinical Research Coordinator	Julie Barenholtz, MSW	Clinical Research Coordinator
Research Dietitian	Corrine Rossi, RD, LDN	Study Dietitian
Diet Technician	Liz Cannell	Diet Technician/Chef
Biostatistician	Henry Feldman	Senior Biostatistician

Collaborators at UMass Boston

Role	Name	Title
Principal Investigator	Laura Hayman, PhD, RN, FAAN	Associate Dean for Research, College of Nursing and Health Sciences
Co-Investigator	Scott Crouter, PhD	Assistant Professor, Department of Exercise & Health Sciences
Co-Investigator	Jessica Whiteley, PhD	Assistant Professor, Department of Exercise & Health Sciences
Nurse Coordinator	Alyson Karakouzian	Nursing Student
Project Manager	Albert Kim	Project Manager
Facility Contact	Carlos Salas, BS	Exercise Physiologist

b. Contact Information

First Name	Last Name	Email	Phone
Tracy	Antonelli	tracy.antonelli@childrens.harvard.edu	857-218-4709
Annette	Baker	annette.baker@cardio.chboston.org	617-355-7579
Julie	Barenholtz	julie.barenholtz@childrens.harvard.edu	
Mark	Berry	mark.berry@childrens.harvard.edu	617-355-0576
Liz	Cannell	elizabeth.cannell@childrens.harvard.edu	
Scott	Crouter	scott.crouter@umb.edu	617-287-7509

Sarah	de Ferranti	sarah.deferranti@cardio.chboston.org	617-355-0955
Laura	Hayman	laura.hayman@umb.edu	617-287-7504
Alyson	Karakouzian	alyson.karakouzia011@umb.edu	
Albert	Kim	albert.kim@umb.edu	
Sarah	Krathwohl	sarah.krathwohl@childrens.harvard.edu	857-218-4741
Stavroula	Osganian	stavroula.osganian@childrens.harvard.edu	857-218-4955
Moriah	Polanco	moriah.polanco@childrens.harvard.edu	857-218-4710
Corrine	Rossi	corrine.rossi@childrens.harvard.edu	
Carlos	Salas	carlos.salas@umb.edu	
Jessica	Whiteley	jessica.whiteley@umb.edu	617-287-3807

III. Recruitment

a. Inclusion and Exclusion Criteria

Main Experiment

Inclusion criteria

- enrolled in grades 3-5
- able to attend GoKids and school-based weekly educational sessions for 12 weeks and complete baseline and follow-up testing
- informed consent from a parent or legal guardian and written assent from the child
- English speaking child
- free from medical conditions that would prohibit exercise as indicated by permission to participate in school physical education, and by review of screening questions with clearance from primary care provider as necessary

Exclusion criteria

- abnormalities on screening/baseline evaluation that could pose a significant risk for exercise
- illness that would limit participation
- plans to move out of the area or change schools in the next 6 months

Sub-Study

Inclusion criteria

- participation in the Main Experiment
- able to attend CHB CTSU for two ~2 hour visits occurring at baseline and follow-up
- informed consent for the Sub-Study from a parent or legal guardian and written assent (from the child)
- HDL <50 mg/dL, TC > 170 mg/dL, blood pressures > 90th percentile for age, gender and height, OR waist circumference > 90th percentile (NHANES)

Exclusion criteria

- as above

b. Recruitment Procedures

Main Experiment

Children will be recruited from Boston Public Schools. Tamara Blake-Canty, the principal of Russell Elementary School has expressed interest in participation and will be instrumental in

guiding recruitment logistics. Recruitment materials briefly describing the Main Experiment and the Sub-Study will be sent home with school children along with a consent form and the screening form. Teachers designated by the principal may contact parents to follow-up regarding enrollment materials. If health concerns are raised by the screening form, physician clearance will be requested from the primary care physician. The child assent form will be completed during his or her first visit to GoKids. Students from the third through fifth grades will be approached to participate. Study activity will occur outside school hours, unless otherwise requested by school officials.

Sub-study

After the baseline assessment for the Main Experiment, eligibility for participation in the Sub-Study will be reviewed. Parents will be contacted by phone to receive Main Experiment lab results; at this time, if the child meets the eligibility criteria for the Sub-Study, the parent and child will be invited to join the Sub-Study. If the family is amenable, a CHB CTSU visit will be arranged prior to the start of physical activity. Consent and assent for the Sub-Study will be collected at the time of the CTSU visit.

c. Screening

Main Experiment

Prior to participation in screening, enrollment forms filled out by parents either at evening sessions at the school or at home will be required. If risks for exercise are discovered on the enrollment form, physician clearance for participation will be required. Study staff will help facilitate this by faxing forms to pediatricians and coordinating the return of these forms.

Sub-study

After the baseline assessment for the Main Experiment, eligibility for participation in the Sub-Study will be reviewed (see Recruitment Procedures, above).

d. Informed Consent

Main Experiment

Informed consent will be obtained by mailing enrollment packets home to parents prior to conducting baseline procedures. Parents may directly contact study staff with any questions regarding information in the consent form.

Assent will be obtained by the study coordinator or PIs prior to conducting baseline procedures at GoKids. The PI or study coordinator will verbally review the assent form, will discuss the study and answer any questions. The child will be instructed that participation is voluntary and that s/he is free to withdraw at anytime.

Sub-study

Informed consent and assent will be obtained by the study coordinator or PIs prior to conducting baseline procedures in the CHB CTSU. The parent or legal guardian or adult participant will be given the consent form to read and a verbal explanation of the study risks and benefits of participation will be provided by the study coordinator or PIs. The PIs will be available to review the study, clarify any areas that are confusing, and answer any questions. The purpose, study duration, procedures, potential risks and benefits, and the subjects' rights will be explained. The subject will be instructed that participation is voluntary, and that his or her medical care or participation in the Main Experiment will not be affected by his or her participation and that s/he is free to withdraw at any time. Asking the subject and parent or legal guardian to explain the study in his/her own words will assess the subject's understanding and autonomy. Each subject will be informed that, if he/she is a CHB patient, access to his/her medical records and trial related

source documents will be granted for research-related monitoring and IRB review, and regulatory inspection. Subjects also will be informed that if abnormal test results are obtained during the screening process that could affect the care they receive from their primary care physician, their tests results will be provided to their physician with patient/parent permission.

e. Baseline Assessment

Main Experiment

All participants will be asked to attend GoKids UMass Boston (Boston, MA) for baseline measurements. During the baseline visit, research personnel will screen for eligibility and obtain a comprehensive assessment of baseline cardiovascular, physical activity, and psychosocial measures under the supervision of trained research personnel and exercise physiologists. Non-fasting finger stick total cholesterol (TC) and high-density lipoprotein (HDL) testing will be offered. If no exclusionary criteria become apparent during baseline measurements, participants will be individually randomized to either the center-based exercise intervention (*GoKids*) or an *Advice-Only* condition for 12 weeks, with a follow-up evaluation at 12 weeks after baseline. See Study Measurements and Data Collection for more information.

Sub-study

All eligible participants will be scheduled for a sub-study visit in the CHB CTSU for baseline measurements. During the baseline visit, research personnel will obtain informed consent and assent for participation in the sub-study. Once consent/assent is obtained, research personnel will complete baseline measurements under the supervision of the sub-study PI. See Study Measurements and Data Collection for more information.

f. Incentives

Main Experiment

Participants will be given a steel water bottle at the end of the baseline visit. Participants will be given a (TBD) at the end of the 12-week visit.

Participants will receive afternoon snacks at every exercise and nutrition education session.

The school will receive (TBD) at the end of the study.

Teachers directly involved in supervising children and contacting parents regarding the study will be compensated for their time.

Sub-study

Participants will be given a $50.00 gift certificate to Best Buy and parking voucher or T pass after completing each study visit (baseline and 12-week visits).

IV. Study Visits

a. Study Visit Schedule

Main Experiment (2010)

Study Event	Jan	Feb	Mar	Apr	May	Jun	Jul	Aug	Sep	Oct	Nov	Dec
Screening		X				X			X			
Baseline			X						X			

12-week assessment						X						X

Sub-study (2010)

Study Event	Jan	Feb	Mar	Apr	May	Jun	Jul	Aug	Sep	Oct	Nov	Dec
Baseline			X						X			
12-week assessment						X						X

b. Intervention Protocol

Following the baseline measurements, participants will be individually randomized into one of the following two treatment conditions: *GoKids* and *Advice-Only*. All participants will receive weekly group nutrition and activity education sessions lasting approximately 30 minutes supported by printed dietary, exercise and motivational advice adapted from existing materials (clinic materials, AHA, CDC and NHLBI materials). The sessions will be taught by UMass student educators trained to deliver a standard message. The *Advice-Only* intervention will consist entirely of these weekly sessions. It was felt to be unethical to offer no support for following a heart-healthy lifestyle to children randomized to the *Advice-Only* condition given the high rate of overweight/obesity and generally poor diet and activity levels in our study population. The *Advice-Only* group will be offered the 12-week GoKids physical activity experience at the close of the trial as an incentive for participation. Children in both groups will receive a small incentive during week 6 and then at the close of the intervention. In addition, classroom and school-wide incentives will be offered, such as healthy lifestyle education sessions for other classes; details will be discussed with the school principal.

The *GoKids* group will receive the GoKids integrated program which uses theory-based behavioral strategies and center-based exercise training. It includes an initial exercise consultation, orientation, and tri-weekly training at *GoKids* Boston. Previous research from GoKids Boston indicates that the energy expenditure from playing interactive video games is comparable to what is expended while walking on a treadmill for the same amount of time. In addition, the use of interactive video games is at a sufficient intensity to engage the participant in MVPA (Bailey et al., 2008). Exercise sessions in the *GoKids* condition will occur 3 times per week lasting 60 minutes each session, for 12-weeks and will include a variety of exercise training activities using the latest interactive exercise games, treadmills, elliptical machines, and strength training equipment located at the UMass facility. All sessions will be supervised by a trained exercise physiologist, with nurse practitioner support available and the classroom teacher present and active with the students.

	Monday	Tuesday	Wednesday	Thursday	Friday
	(60 minutes)	(30 minutes)	(60 minutes)		(60 minutes)
***GoKids* (intervention)**	Exergaming at GoKids	Group session in classroom	Exergaming at GoKids	(no activity)	Exergaming at GoKids
***Advice-Only* (control)**		Group session in classroom		(no activity)	

c. Randomization Procedures

Staff at the Clinical Research Program (CRP) of CHB will run the randomization software based on the specifications provided by the Principal Investigator (PI). The randomization software (SciRAN) was developed by the Clinical Research Information Technology (CRIT) in collaboration with CRP Biostatistics Core. SciRAN is password protected and access is restricted to designated CRIT and CRP personnel only. The system is designed to a) generate unique and unpredictable random assignments to treatment groups; b) provide users with an audit trail of the process including a verifiable link between subject ID and treatment assignment; and c) provide standard documentation tools.

Key randomization specifications for this pilot study include the following:

- One hundred (100) subjects randomized at CHB, 50 to each of the two treatment arms.
- Children in third through fifth grade will be randomly assigned to one of the following two treatment arms after eligibility is confirmed:
  - **Group A:** Nutrition education + exer-gaming sessions
  - **Group B:** Nutrition education
- Stratification: normal weight and overweight/obese participants
- The PI will be blind to treatment assignment and block sizes.

Prior to running the randomization program for the study, Dr. de Ferranti confirmed the randomization specifications by completing the Randomization Checklist. Dr. Feldman, Senior CRP Biostatistician, approved the specifications and prescribed the randomization block sizes. Finally, the CRP Data Manager ran the randomization program inputting the approved specification and produced the following randomization documents:

- The Master Randomization Lists: One list per school site: the Master Randomization list includes randomization identifiers, corresponding treatment assignments and randomization blocks.
- The Investigator's Randomization List, which is a 'blinded' version of the Master Randomization List, i.e., absent the treatment assignments and randomization blocks. The investigator's Randomization List will be delivered to the PI.

d. Study Close-out

Study close-out activities occur at the completion of the study or in the case of early termination. Early termination of the study may occur if funding is stopped or if there are serious adverse events.

Upon completion or early termination of the study, the PI must:

- Confirm the completion of all case report forms
- Review and resolve all data queries
- Fill out and submit a Completion Form to CCI
- Complete a final report

V. Study Measurements and Data Collection

a. Administering Surveys/Questionnaires

Main Experiment

Enrollment Form

- Completed by a parent/guardian with other enrollment materials, including informed consent documents
- Includes demographic and medical history that will be entered into study database (date of birth, gender, race/ethnicity, household, health history, healthcare source, employment status, education level, free/reduced price school lunch)

Survey (Diet and Physical Activity)

- Completed by participant at baseline and 12-week visits

Modified Harter Scale

- Completed by participant at baseline and 12-week visits
- See the Appendices for Question-by-Question guidelines

Selected TAAG Questions

- Completed by participant at baseline and 12-week visits
- See the Appendices for Question-by-Question guidelines

Satisfaction Surveys

- Daily Activity Satisfaction Survey
  - Completed by participant at baseline and 12-week visits
- 12-week Child Satisfaction Survey
  - Completed by participant at 12-week visit
- 12-week Administrator Satisfaction Survey
  - Completed by principal after 12-week visit

Sub-study

Dietary Intake Assessment (24-Hour Recall)

- Participants will complete one 24-hour dietary recall interview at baseline and 12-week visits.
- The face-to-face interview will be conducted by the CTSU research dietician using the interactive Nutrient Data System (NDS) (Nutrition Coordinating Center, University of Minnesota, Minneapolis, MN), following standard NDS protocols that have been tested in youth.
- Three-dimensional food models will be used to quantify portion sizes.
- Data will be entered directly into a laptop computer.

b. Procedures and Examinations

Please see the Appendices for the following protocols:

Main Experiment

- Height, Weight, and Bioelectrical Impedance Assessment (BIA)
- Waist Circumference
- Blood Pressure
- FitnessGram Pacer
- Accelerometry

Sub-study

- Height and Weight
- EndoPAT (Vascular Reactivity)
- DXA (Body Composition and Density)
- Blood Pressure
- Indirect Calorimetry
- Left Ventricular Mass by Echocardiogram

c. Sample Collection

Please see the Appendices for the following protocols:

Main Experiment

- Cholestech Total Cholesterol/HDL

Sub-study

- Fasting Labs
- Urine Pregnancy Test

d. Case Report Form Completion

Main Experiment
Enrollment

Form	Filled out by…	Recruitment/ Enrollment
Enrollment Form	Parent	x
Liability Agreement	Parent	x
GoKids Facility Consent	Parent	x
Medical Release Form	Parent	x
Study Consent Form	Parent	x
Physician's Clearance	EXCEL Study Staff	x

Baseline and 12-week Assessments

Form #	Form	Completed by…	Baseline Visit (at GoKids)	Inter-vention	12-week Visit	As Needed
	Participation Agreement	Child	x			
	GoKids Assent Form	Child	x			
	Study Assent Form	Child	x			
2	Survey	Child	x		x	
3	Modified Harter Scale	Child	x		x	
4	Selected TAAG Scales	Child	x		x	
5	Pacer Form		X		x	
1	Form 1: Anthropometric and Lab Data	EXCEL Study Staff	x		x	
6	Accelerometry	Child	(1 week after baseline)		x	
	Daily Satisfaction Survey	Child		x		
	12-week Child Satisfaction Survey	Child			x	
	Administrator Satisfaction Survey	Principal			x	
	Attendance	Computer printout	x	x	x	
7	Adverse Events Tracking	EXCEL Study Staff				x
8	Trial Termination Form	EXCEL Study Staff			x	

Sub-study

Form #	Form	Screening	Baseline (at CHB)	12-week	As Needed
2	Eligibility	x			

3	Study Visits		x	x	
4	Clinical Labs		x	x	
6	24-Hour Dietary Recall		x	x	
7	Trial Termination			x	
5	Adverse Events Tracking				x

e. Study Monitoring

Laboratory parameters will be reviewed regularly, at least monthly, by Drs. de Ferranti and Osganian. Exercise metrics will be reviewed at least monthly by Dr. Crouter.

VI. Adverse Event Reporting

a. Risks and Discomforts

Main Experiment

Risks and discomforts are expected to be minimal in children enrolled in the Main Experiment, as the intervention will involve physical activity aimed at children. The fingerstick TC and HDL testing will be optional. Minimal risk due to injury may be associated with the exercise intervention, but no more than a school-based physical education class. Questionnaires occasionally can be distressing. Support will be available from GoKids staff experienced in interacting with children.

Sub-study

Risks and discomforts for the Sub-Study will involve EndoPAT vascular testing, which we have shown is well tolerated in adolescents (Tierney 2009) and a fasting blood draw, which is not generally too onerous for children this age. In the CTSU, blood is drawn by trained nurses experienced in pediatric phlebotomy.

b. Reporting and Follow-up Procedures

Main Experiment

Adverse events associated with the Main Experiment will be immediately reported to the PIs at UMass Boston and CHB. An Adverse Event Tracking Form will be completed and followed up until determined by the PI as resolved or unresolved.

If the event is “reportable”, the Adverse Event Tracking Form will be submitted to the UMass Boston IRB. Reportable adverse events are defined by the UMass IRB as:

Sub-study

Adverse events associated with the Sub-study will be immediately reported the PI at CHB. An Adverse Event Tracking Form will be completed and followed up until determined by the PI as resolved or unresolved.

If the event is “reportable”, the Adverse Event Tracking Form will be submitted to the CHB CCI. Reportable adverse events are defined by the CHB CCI as:

- Adverse events that are also unanticipated problems involving risks to subjects or others
- Unanticipated events that do not involve actual harm to subjects or others, but which place research subjects or others at risk of harm that was not previously anticipated.
- Any death of a study subject thought to be related or possibly related to the research associated interventions.

VII. Data Management Procedures

a. Overview of Data Flow

Main Experiment data will be collected at UMass GoKids site. Sub-Study data will be collected at CHB. All data will computer entered and stored at CHB. Case report forms will be transported to CHB on a regular basis using a locked document box accompanied by research personnel. On CRFs, patients will be indentified only by a study ID. Identified information will be stored at CHB with a study log maintained at UMass and also at CHB. Randomization and data entry will occur at CHB.

b. Data Entry Procedures

Upon receipt of data forms, Data Coordinator Moriah Polanco will enter forms into SPSS databases on the secure study folder. Entered forms will be stamped to denote completion and will be filed in locked cabinets at CHB.

c. Form Completion Tracking

Data Coordinator Moriah Polanco will track all received and entered forms per participant per visit for both the Main Experiment and Sub-study in an Excel spreadsheet. The project manager will review the form completion and entry tracking sheet for completeness at 4 weeks after receipt of completed forms.

d. Data Entry Error Resolution and Editing Procedures

Visual review 10% of database entries compared to case report forms or source documentation (for example, lab data from printouts) will be completed by the project manager. All discrepancies will be noted in an Excel spreadsheet. Data entry errors will be corrected by the Data Coordinator and error resolution will be documented in the same Excel spreadsheet.

VIII. Quality Control

a. Training and Certification

i. Human Subjects Training

All study personnel must maintain current human subjects training certification and HIPAA certification. PIs at CHB and UMass must monitor the status of current certification and maintain files of current certificates for each investigator and study staff member.

ii. Standardized Methods of Data Collection

All study personnel must obtain appropriate training to be document in a study Training Log, indicating trainer and date, prior to data collection.

iii. Standardized Delivery of Nutrition Education Curriculum

Dietitian Corrine Rossi and Diet Technician Liz Cannell are trained nutrition educators. Corrine will be the primary teacher; Liz will monitor lessons for consistency between intervention and control groups to ensure standardized delivery.

b. Laboratory Quality Control

Main Study
Cholestech – Quality Control measures as outlined by the Cholestech User Manual will be followed whenever the machine is moved as well as on the day prior to use of the machine.

c. Communication Procedures

The Children's Hospital Boston will coordinate bi-weekly research team meetings during study initiation and monthly team meetings during data collection and intervention stages. The Lead Recruiter, Project Manager, and Principal Investigator will have weekly contact by phone or email regarding recruitment. Monthly team meetings will be coordinated during data collection, or as needed. Meetings will be alternately held at UMass Boston and Children's Hospital Boston. If a physical meeting is not feasible, a conference call will be scheduled.

Adverse events, protocol deviations, or complaints associated with the Main Experiment will be immediately communicated to both Principal Investigators at UMass Boston and Children's Hospital Boston. Adverse events, protocol deviations, or complaints associated with the Sub-study will be immediately communicated to the Principal Investigator at Children's Hospital Boston. Reporting of adverse events to the IRB should follow IRB reporting procedures listed above.

Investigators and research teams from UMass Boston and Children's Hospital Boston will maintain clear email communication to

IX. References

American Heart Association (2005). Overweight in Children and Adolescents: Pathophysiology, Consequences, Prevention, and Treatment. *Circulation*, 111, p.1999-2012.

An,JYA et al. (2009) Web-based weight management programs for children and adolescents: A systematic review of randomized controlled trial studies. *Advances in Nursing Science* 32(3), p. 222-40.

Bailey, B. et al. (2008) Energy cost of ExerGaming in adolescents (Abstract). *Obesity*, 16 (Supplement 1), p. S77-S78.

Berkey CS et al. (2000) Activity, dietary intake, and weight changes in a longitudinal study of preadolescent and adolescent boys and girls. *Pediatrics*. 105(4), p. E56.

Caranti DAde Piano A et al. (2007). Short- and long-term beneficial effects of a multidisciplinary therapy for the control of metabolic syndrome in obese adolescents. *Metabolism*, 56(9),1293-300.

Churilla JR, Zoeller RF. (2008). Physical activity and the metabolic syndrome: A review of the evidence. *American Journal of Lifestyle Medicine*, 2(2), p. 118-125.

Daley AJ. (2009) Can Exergaming Contribute to Improving Physical Activity Levels and Health Outcomes in Children? *Pediatrics,* 124;763-771 e-published ahead of print Jul 13, 2009.

Dishman RK, et al., (2009) Validity of Social-Cognitive Measures for Physical Activity in Middle-School Girls. *J Pediatr Psychol*. 2009 ePub 2009 May 25

Ferguson, M.A., et al., (1999) Effects of exercise training and its cessation on components of the insulin resistance syndrome in obese children. *Int J Obes Relat Metab Disord*, 23(8), p. 889-95.

Freedson PD, et al, (2005) Calibration of accelerometer output for children. *Med Sci Sports Exerc*, 37(11 Suppl), p. S523-30.

Harter, S. (1985) Manual for the self-perception profile for children. Denver: University of Denver.

Hayman LL, Reineke PR. (2006) Promoting cardiovascular health in children and adolescents. *Journal of Cardiovascular Nursing*. 21(4), 269-275.

Jolly K, Taylor RS et al.(2006). Home-based cardiac rehabilitation compared with centre-based rehabilitation and usual care: a systematic review and meta-analysis. *International Journal of Cardiology*, 111, 343-351**.**

Klijn PH, et al. (2007). Aerobic exercise in adolescents with obesity: preliminary evaluation of a modular training program and the modified shuttle test. *BMC Pediatrics*, Apr 19;7:19*.*

Lohman, TG, Roche AF, Martorell R. (1988). *Anthropometic Standardization Reference Manual*.

Morrison JA et al. (2008). Metabolic syndrome in childhood predicts adult metabolic syndrome and type 2 diabetes 25 to 30 years later. *J Pediatr*. 152, 201-206.

Norman G. et al. (2007). A review of eHealth interventions for physical activity and dietary behavior change. *Am J Prev Med*, 33, 336-345.

Reinehr T, et al (2009). Lifestyle intervention in obese children is associated with a decrease of the metabolic syndrome prevalence. *Atherosclerosis*, (doi:10.1016),

Rockett HR et al (1997) Validation of a youth/adolescent food frequency questionnaire. *Prev Med.* Nov-Dec;26(6):808-16.

Rosenberg, M. (1965). Society and the adolescent self-image. Princeton, NJ: Princeton University Press.
Steinberger J et al. (2009) Progress and challenges in metabolic syndrome in children and adolescents. *Circulation*.;119(4):628-47. Epub Jan 12

Strong, W.B., et al., (2005) Evidence based physical activity for school-age youth. *J Pediatr* 146(6): p. 732-7

Selamet Tierney ES et al. Endothelial Pulse Amplitude Testing: Feasibility and Reproducibility in Adolescents. *J Pediatr.* 2009;154(6):901-5. Epub 2009 Feb 12

Troped PJ et al. (2007) Reliability and validity of YRBS physical activity items among middle school students. *Med Sci Sports Exerc*. Mar;39(3):416-25.

Trost SG. (2007). State of the art reviews: Measurement of physical activity in children and adolescents. *American Journal of Lifestyle Medicine*.

Whiteley, J. et al. (2008). Using the Internet to Promote Physical Activity and Healthy Eating in Youth*. American Journal of Lifestyle Medicine*, 2 (2),159-177.
