# Supplementary material for: Effect on Physical Activity of a Randomized Afterschool Intervention for Inner City Children in 3rd to 5th Grade
Source: PLoS One. 2015 Oct 28;10(10):e0141584. doi: 10.1371/journal.pone.0141584 (PMC4624808; doi:10.1371/journal.pone.0141584)
Supplement: S1 Text — (PDF) [file pone.0141584.s004.pdf]

UMass Boston - Go Kids Excel Program
Nutrition Education Workshop
Children's Hospital Boston

## WORKSHOP SUMMARY

**WHO:** Children in 3rd-5th grade enrolled in UMass Boston Go Kids Excel Program

**WHAT:** 30 minute nutrition education workshops (15 minutes of education and 15 minutes activities) once per week for ten weeks

**WHEN:** TBA

**WHERE:** UMass Boston Go Kids Fitness Center

**WHY:** To teach children the importance of making healthful food choices

## WORKSHOP TOPICS

| Workshop Topic | Lesson | Activity | Handout |
|---|---|---|---|
| Week 1 – Food Guide Pyramid – Grains | | | |
| Week 2 – Food Guide Pyramid – Vegetables | | | |
| Week 3 – Food Guide Pyramid – Fruit | | | |
| Week 4 – Food Guide Pyramid – Dairy | | | |
| Week 5 – Food Guide Pyramid – Meat and Beans | | | |
| Week 6 – Food Guide Pyramid – Oils (Fats) | | | |
| Week 7 – Beverages and Snacks | | | |
| Week 8 – Dining Out | | | |
| Week 9 – Mindful Eating | | | |
| Week 10 – Goal Setting | | | |

## Week 1 – Food Guide Pyramid – Grains

- Use the USDA Food Guide Pyramid to explain recommendations for food group servings (grains).
- Demonstrate the difference between refined and whole grains
- Provide examples of refined and whole grains and serving sizes (demonstrate with food models)
- Provide a snack as well as food samples (whole grains)
- Activity

## Week 2 – Food Guide Pyramid – Vegetables

- Use the USDA Food Guide Pyramid to explain recommendations for food group servings (vegetables).
- Provide examples of starchy/non-starchy vegetables and serving sizes(demonstrate with food models)
- Provide a snack as well as food samples (vegetables)
- Activity

## Week 3 – Food Guide Pyramid – Fruit

- Use the USDA Food Guide Pyramid to explain recommendations for food group servings (fruit).
- Provide examples of various types of fruit and serving sizes (demonstrate with food models)
- Provide a snack as well as food samples (fruit)
- Activity

## Week 4 – Food Guide Pyramid – Dairy

- Use the USDA Food Guide Pyramid to explain recommendations for food group servings (dairy).
- Provide examples of various types of dairy foods and serving sizes(demonstrate with food models)
- Provide a snack as well as food samples (dairy)
- Activity

## Week 5 – Food Guide Pyramid – Meat and Beans

- Use the USDA Food Guide Pyramid to explain recommendations for food group servings (meat and beans).
- Provide examples of various types of meat/bean foods and serving sizes(demonstrate with food models)
- Provide a snack as well as food samples (meat and beans)
- Activity

## Week 6 – Food Guide Pyramid – Oils (Fat)

- Use the USDA Food Guide Pyramid to explain recommendations for food group servings (oils).
- Provide examples of various types of dairy foods and serving sizes (demonstrate with food models)
- Provide a snack as well as food samples (healthful fats)
- Activity

## Week 7 – Beverages and Snacks

- Explain why is it important to eat small meals/snacks throughout the day
- Demonstrate balanced snack choices using food models
- Review best beverage choices (use sugar models to demonstrate amount of sugar in various beverages)
- Provide a snack
- Activity

## Week 8 – Dining Out

- Explain why is it important to make healthful food choices when eating at a restaurant
- Demonstrate restaurant portion sizes using food models
- Review common chain restaurant menus to look for the healthiest options
- Provide a snack
- Activity

## Week 9 – Mindful Eating

- Explain the term "mindful eating". What does it mean to be "mindful"?
- Demonstrate the technique of "eating mindfully" (use all senses to fully appreciate the experience of eating)
- Provide a snack as well as food for the activity
- Activity

## Week 10 – Goal Setting

- Explain the importance of setting goals
- Explain the difference between a short term and long term goals
- Demonstrate the S.M.A.R.T technique for setting goals
- Provide a snack
- Activity

# UMass Boston: GoKids
# Lesson 1 – Food Guide Pyramid - Grains

Teacher: TBA
Target Audience: Children in grades 3-5 enrolled in GoKids
Date: TBA
Time: 30 minutes

## Timeline:

- Welcome – 1-2 minutes
- Education – 15 minutes
  - What are grains? Use FGP to explain recommendations and serving sizes for grains
  - Refined vs. Whole grains
  - What do whole grains taste like?
  - What is a serving and how many servings do I need each day?
- Activity – 10-15 minutes
- Questions

## Materials needed:

- Food sample: whole grains (i.e. multigrain Puffins cereal)
- Plates, cups, napkins, utensils, snack
- Activity 1: Grains
- Handout 1: Serving sizes for grains

## Food models needed:

- Food models for grains (i.e. cereal boxes, bread bags, 2D, 3D food models)

## Welcome Message: Before beginning, distribute the snack and beverage.

Hello. My name is_______________. I am a nutritionist. **QUESTION: Does anyone know what a nutritionist does?** (allow a few moments for responses) A nutritionist is someone who teaches people about food and health. For the next few weeks I am going to be talking to you about tasty foods that are also good for you.

Before we start our lesson for today, I would like to have everyone in the class stand up and stretch!
Ask students to stand up and follow a series of guided stretches (upper body stretches and taking deep breaths)

## Education:

Today I would like to talk with you about choosing healthy foods. We all eat food because food gives us energy.

**QUESTION: What does energy do for us?** Energy gives us the "power" that we need to live.

**QUESTION: What kinds of activities do we need energy for?** Walking, talking, playing, running, learning, dancing. We need energy for everything we do!

**QUESTION: Does anyone know where the energy comes from in food?** It comes from little tiny things called nutrients. Nutrients are in all of the foods on the Food Guide Pyramid.

**Question:** Who has seen the Food Guide Pyramid before? (Show the large poster/image of the FGP).

The different colored sections represent the different food groups. For the next few weeks we are going to talk about the Food Guide Pyramid. Today we will talk about grains.

---

**QUESTION: What are grains?** (3)

Grain give us tons of instant energy and make up the base of the Food Guide Pyramid. There are two types of grains. They are called refined or whole grains.

**QUESTION: Can anyone give me an example of a refined or whole grain? (use real food to show examples of refined and whole grains. i.e. white rice, cereal boxes, whole wheat bread)**

Whole grains -- Examples include:

- 100% whole-wheat bread
- oatmeal
- brown rice

Refined grains – Examples include:

- white bread
- white rice
- Rice Krispies cereal

---

**QUESTION: What is the difference between whole and refined grains?**

- Whole grains are usually brown in color and are full of vitamins, minerals, and fiber
- Refined grains are usually white in color and do not have the vitamins, minerals, and fiber that whole grains have

**QUESTION: How do you know how many servings of grain you need in a day?** The FGP
Let's take a look at this handout on grains and the food models that I brought to see the different serving sizes.

Explain to the class that children between the ages of ___generally need ___servings of grain each day.

Because whole grains are a healthier choice, a good goal is to make half your grain servings, whole grains.

## Activity:

Now I would like to take a few minutes to do a fun activity!

Activity: Red Light, Green Light using the scripted questions/answers on the instructor activity guide. The questions/answers for this game are related to grains and are a summary of the information presented in the lesson.

## Questions:

**QUESTION: What questions can I answer for you about today's lesson?**

## Resources:

(1) http://www.mypyramid.gov/pyramid/index.html

(2) http://aappolicy.aappublications.org/cgi/content/full/pediatrics%3B107/5/1210

(3) http://kidshealth.org/kid/stay_healthy/food/carb.html

# Grains

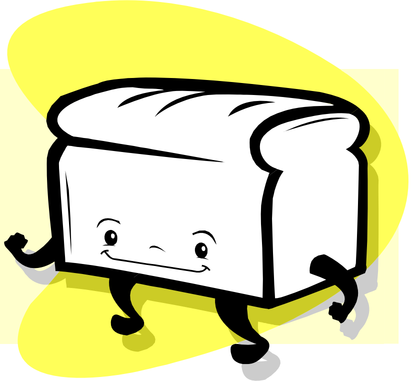

What counts as one serving of **grain**? 1 serving = 1 oz
How many servings of **grain** do I need each day? ~ 6 oz/day

| Whole Grains | 1 Serving = 1 oz |
|---|---|
| 100% whole wheat bread | 1 regular slice |
| Whole wheat English Muffin | ½ muffin |
| Oatmeal | ½ cup cooked or 1 packet |
| Bulgur or barley | ½ cup cooked |
| Brown rice | ½ cup cooked |
| Whole grain crackers | 5 whole wheat crackers |
| Cold cereal (high fiber) | 1 cup flakes |
| Popcorn | 3 cups, popped |

| Refined Grains | 1 Serving = 1 oz |
|---|---|
| White bread | 1 regular slice |
| Pancakes | 1 pancake (4” diameter) |
| Bagels | 1 mini bagel |
| Cold cereal (low fiber) | 1 cup flakes |
| White rice | ½ cup cooked |
| Pasta | ½ cup cooked |
| Muffins | 1 small (2 ½” diameter) |
| Cornbread | 1 small piece (2 ½ x 1 ¼ x 1 ¼ ) |

Activity: Red Light, Green Light

| Grains | | |
|---|---|---|
| # | Question | Answer |
| 1 | True or false: white rice is a whole grain? | False |
| 2 | True or false: refined grains have more fiber than whole grains? | False |
| 3 | Name the main ingredient in whole wheat bread. | Wheat |
| 4 | True or false: fiber is important for good health? | True |
| 5 | True or false: Food gives us the calories (energy) we need to live, study, work, and play? | True |
| 6 | Name a refined grain food that is part of lasagna. | Pasta |
| 7 | Name a whole grain. | Brown rice, whole wheat bread, barley, oats |
| 8 | How many servings of grain do kids your age need in one day? | 6 |
| 9 | Name a whole grain breakfast item. | Whole wheat toast, oatmeal, etc. |
| 10 | What % of your grains should be whole? | 50% |


Adapted from: Eat Well and Keep Moving
Copyright © 2001 by Lilian W.Y. Cheung, Steven L. Gortmaker, and Hank Dart

# UMass Boston: GoKids
# Lesson 2 – Food Guide Pyramid – Vegetables

Teacher: TBA
Target Audience: Children in grade 3-5 enrolled in GoKids
Date: TBA
Time: 30 minutes

## Timeline:

- Welcome – 1-2 minutes
- Education – 15 minutes
    - What are vegetables? Use FGP to explain recommendations and serving sizes for vegetables
    - Where and how do they grow?
    - What do they taste like?
    - What is a serving and how many servings do I need each day?
- Activity – 10-15 minutes

## Materials needed:

- Food sample: raw vegetables
- Plates, cups, napkins, utensils, snack
- Activity 2: Vegetables
- Handout 2: Vegetables

## Food models needed:

- 2D and 3D food models

## Welcome Message: Before beginning, distribute the snack and beverage.

Hello. **QUESTION: How are you doing this week?**

Before we start our lesson for today, I would like to have everyone in the class stand up and stretch!
Ask students to stand up and follow a series of guided stretches (upper body stretches and taking deep breaths)

## Education:

Today I would like to talk to you about vegetables. Start the discussion by asking students to identify their favorite types of vegetables.

**QUESTION: What are some of your favorite vegetables?**

**QUESTION: What are vegetables?** Vegetables are a big part on the Food Guide Pyramid and that means that we need several servings each day to make our plate balanced. Vegetables are the parts of plants that you can eat! These parts include stems, roots, flowers, and leaves of plants.

Prompt with questions, such as: **QUESTIONS: When do you eat vegetables? Why do you eat vegetables? Are all vegetables green?**

---

Let's take a look at this handout on vegetables and their serving sizes, then we will sample a few different types of raw vegetables. There are thousands and thousands of different varieties of vegetables in the world. It is amazing! Vegetables come in all different colors and shapes. It is best to eat a variety! There are many different ways to categorize vegetables and today we are going to look at one of those ways. Vegetables are so good for you! They are loaded with vitamins, minerals, and fiber to keep you healthy and feeling full. Kids your age need 2 cups of vegetables per day to stay healthy and strong.

Demonstrate portion sizes using the food models and handout as a reference.

## Root and Tuber Vegetables

**QUESTION: Can anyone name some root or tuber vegetables (vegetables that grow under ground)?** Radishes, carrot, parsnip, beets, turnip, and potatoes, sweet potatoes, yams, jicama, water chestnuts
**QUESTION: Where does this type of vegetable grow? On a tree or vine, etc.?** Root and tuber vegetables – the edible part of these vegetables (the root) grows underground. These vegetables are rich in nutrients!

---

## Bulb and stem vegetables

**QUESTION: Can anyone name some bulb or stem vegetables?** Asparagus, celery, garlic, onions, leeks, shallots, scallions
**QUESTION: Where does this type of vegetable grow? On a tree or vine, etc.?** In general, these vegetables grow above the ground in stalks or below the ground on bulbs.

---

## Podded vegetables

**QUESTION: Can anyone name some podded vegetables?** Chickpeas, peas, snowpeas, lentils, green beans, wax beans
**QUESTION: Where does this type of vegetable grow? On a tree or vine, etc.?** In general, these vegetables grow above ground on plants inside of pods.

---

## Fruit and flowering vegetables

**QUESTION: Can anyone name some fruit and flowering vegetables?** Broccoli, eggplant, cucumbers, zucchini, cauliflower, sweetcorn
**QUESTION: Where do these types of vegetables grow? On a tree or vine, etc.?** In general, these vegetables grow on plants above ground

## Leafy and salad green vegetables

**QUESTION: Can anyone name some leafy and salad green vegetables?** Cabbage, lettuce, spinach, mixed greens, endive, collard greens, kale, mustard greens
**QUESTION: Where does these types of vegetables grow? On a tree or vine, etc.?** In general, these vegetables grow above ground

---

## Sea vegetables

**QUESTION: Can anyone name some sea vegetables?** Sea kale, sea lettuce
**QUESTION: Where does this type of vegetable grow? On a tree or vine, etc.?** In general, these vegetables grow in the ocean!!
**QUESTION: Can you think of a type of food that might have seaweed in it?** Sushi

## Activity:

Now I would like to take a few minutes to do a fun nutrition activity about vegetables!

Distribute the activity to all students. Do the activity as a class. Have the students write their answers in the space provided once you have read the questions aloud.

As an alternative activity, you may choose to play "hangman" with the class using vegetable names as the guessing words.

## Questions:

**QUESTION: What questions can I answer for you about today's lesson?**

## Resources:

(1) http://www.mypyramid.gov/pyramid/index.html

(2) http://aappolicy.aappublications.org/cgi/content/full/pediatrics%3B107/5/1210

(3) http://kidshealth.org/kid/stay_healthy/food/carb.html

(4) http://www.foodsubs.com/FGVegetables.html

# Vegetables

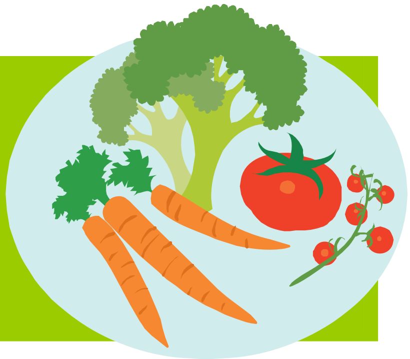

What counts as one serving of **vegetables**? 1 cup
How many servings of **vegetables** do I need each day? 2 cups

| Vegetable | Serving = 1 cup |
|---|---|
| **Root and Tuber Vegetables** | |
| Carrots | 1 cup cooked or 12 baby carrots, raw |
| Potatoes | 1 cup mashed or 1 medium baked |
| Radishes | 1 cup raw |
| Sweet Potatoes | 1 cup mashed or 1 medium baked |
| **Bulb and Stem Vegetables** | |
| Celery | 1 cup chopped or raw |
| Onions | 1 cup chopped or raw |
| Garlic | 1 cup chopped or raw |
| Scallions | 1 cup chopped or raw |
| **Podded Vegetables** | |
| Peas | 1 cup cooked |
| Green beans | 1 cup cooked or raw |
| Snow peas | 1 cup cooked |
| Edamame | 1 cup cooked |
| **Fruit and Flowering Vegetables** | |
| Broccoli | 1 cup florets |
| Cauliflower | 1 cup florets |
| Eggplant | 1 cup cooked |
| Corn | 1 cup or 1 ear (8-9" long) |
| **Leafy Green Vegetables** | |
| Spinach | 2 cups raw, spinach |
| Lettuce | 2 cups raw, lettuce |
| Collard greens | 1 cup cooked collard greens |
| Cabbage | 1 cup cooked cabbage |
| **Sea Vegetables** | |
| Seaweed | 2 cups raw |

Name:______________________________________Date:__________________

| Vegetables | | |
|---|---|---|
| # | Question | Answer |
| 1 | Which tends to have more nutrients? | A. Cooked vegetables<br>B. Raw vegetables |
| 2 | Name two green leafy vegetables. | Spinach, kale |
| 3 | True or false: frozen vegetables are not as good for you as fresh? | False |
| 4 | What type of lettuce is more nutritious? | A. Dark green<br>B. Light green |
| 5 | Name a vegetable that grows in a pod. | Peas |
| 6 | Name a vegetable that grows a "head". | Cabbage, lettuce, cauliflower |
| 7 | Name a blub or stem vegetable. | Celery or onions |
| 8 | What is your favorite vegetable? | ______________________ |
| 9 | Name a root vegetable that is orange and is good for eyesight. | Carrots |
| 10 | Name a flowering vegetable that you might be served in your school cafeteria. | Cauliflower or broccoli |

Go Kids Week 2 Vegetables
Adapted from: Eat Well and Keep Moving
Copyright © 2001 by Lilian W.Y. Cheung, Steven L. Gortmaker, and Hank Dart

# UMass Boston: GoKids
# Lesson 3 – Food Guide Pyramid – Fruit

Teacher: TBA
Target Audience: Children in grade 3-5 enrolled in GoKids
Date: TBA
Time: 30 minutes

## Timeline:

- Welcome – 1-2 minutes
- Education – 15 minutes
  - What is fruit? Use FGP to explain recommendations and serving sizes for various types of fruit
  - Where and how does it grow?
  - What does it taste like?
  - What is a serving and how many servings do I need each day?
- Activity – 10-15 minutes

## Materials needed:

- Food sample: fresh fruit salad
- Plates, cups, napkins, utensils, snack
- Activity 3: Fruit
- Handout 3: Fruit

## Food models needed:

- 2D and 3D food models

## Welcome Message:

Hello. **QUESTION: How are you doing this week?**

Before we start our lesson for today, I would like to have everyone in the class stand up and stretch! Ask students to stand up and follow a series of guided stretches (upper body stretches and taking deep breaths). Now I would like to have everyone in the class go around and tell us about a new food that you ate last week. **QUESTION: What did you like or not like about this food?**

## Education:

Today I would like to talk to you about fruit. I would like to start by doing a quick activity. **QUESTION: Would that be alright with you?**

Close your eyes and picture an orange in your mind. Imagine its shape and color. Think about how it feels in your hands and what it smells like. Keep your eyes closed as you say some words that describe the picture of the orange in your mind. Have students open their eyes. Show them a real orange and let them smell it and touch it. Talk about how their mind-picture of the orange is similar to and different from the real orange.

Expand the discussion by asking students to identify their favorite types of fruits. **QUESTION: What are some of your favorite fruits?**

**QUESTION: What is fruit?** (Fruit is a part of a plant that has the seeds in it.) Encourage students to talk about what the word fruit means.

Prompt with questions, such as: **QUESTIONS: When do you eat fruit? Why do you eat fruit? Is all fruit sweet? Is all fruit small? What does it mean when fruit is "in season?"**

---

Let's take a look at this handout on fruit and serving sizes, then we will sample a few different types of fruit. There are thousands and thousands of different varieties of fruit in the world. It is amazing! There are many different ways to categorize fruits and today we are going to look at one of those ways.

Citrus fruit – Citrus fruits have rinds that surround pulp that's tart, juicy, and rich in vitamin C
**QUESTION: Can anyone name some citrus fruits?** Oranges, grapefruit, tangerines, clementines, tangelos, limes, lemons, kumquats, etc.
**QUESTION: Where does this fruit grow? On a tree or vine, etc.?** In general these fruits grow on trees.

---

Berries - Berries are the delicious and often fragile fruits that are colorful, easy to prepare, good for you.
**QUESTION: Can anyone name some berries?** Blueberries, strawberries, raspberries, blackberries, gooseberries, currants, boysenberries, cranberries, lingonberries, loganberries, kiwi, grapes
**QUESTION: Where does this fruit grow? On a tree or vine, etc.?** In general these fruits grow on vines or bushes.

---

Stone fruit (have pits) – arrive in the summer, and don't become sweeter after they're picked.
**QUESTION: Can anyone name some stone fruit?** Apricots, cherries, peaches, nectarines, and plums
**QUESTION: Where does this fruit grow? On a tree or vine, etc.?** In general these fruits grow on trees.

---

Tropical fruit
**QUESTION: Can anyone name some tropical fruit?** Bananas, coconut, mango, papaya, guava
**QUESTION: Where does this fruit grow? On a tree or vine, etc.?** In general these fruits grow on trees in tropical (warm) areas of the world.

---

Melons
**QUESTION: Can anyone name some melons?** Cantaloupe, honeydew, watermelon,
**QUESTION: Where does this fruit grow? On a tree or vine, etc.?** In general these fruits grow on vines.

---

Pome fruit – comes from a flowering plant
**QUESTION: Can anyone name some pome fruits?** Apples, pears, quince
**QUESTION: Where does this fruit grow? On a tree or vine, etc.?** In general these fruits grow on trees. They grow from flowering plants.

Fruit vegetables – usually fruit is sweet in taste, but certain foods that we consider vegetables are actually categorized as fruits.
**QUESTION: Can anyone name some fruit vegetables ?** tomatoes, eggplants, winter squash, cucumbers, sweet peppers, olives, avocados, and tomatillos.
**QUESTION: Where does this fruit grow? On a tree or vine, etc.?** In general these fruits grow in many different settings. Some grow on trees, others on vines or stalks.

## Activity:

Now I would like to take a few minutes to do a fun nutrition activity about fruit!

Distribute the handout to all students. Do the activity as a class. Have the students write their answers in the space provided once you have read the questions aloud.

Discuss the handout previously distributed (fruit list). Ask the students to take the list home and over the next week their assignment is to try at least two new fruits that they have never eaten.

## Questions:

**QUESTION: What questions can I answer for you about today's lesson?**

## Resources:


(1) http://www.mypyramid.gov/pyramid/index.html

(2) http://aappolicy.aappublications.org/cgi/content/full/pediatrics%3B107/5/1210

(3) http://kidshealth.org/kid/stay_healthy/food/carb.html

(4) http://www.foodsubs.com/FGFruit.html

# Fruit

## What counts as one serving of **fruit**?

| **Fruit** | **Serving = 1 cup** |
|---|---|
| Citrus fruit | |
| Oranges | 1 large, 1 cup sections |
| Grapefruit | 1 medium grapefruit, 1 cup sections |
| Tangerines | 1 tangerine, 1 cup sections |
| Berries | |
| Blueberries | 1 cup berries |
| Strawberries | 1 cup berries, 8 whole strawberries |
| Raspberries | 1 cup berries |
| Stone fruit (with a [illegible] | |
| Peaches | 1 large, 1 cup sliced/chopped |
| Cherries | 1 cup whole cherries |
| Nectarines | 1 large nectarine |
| Tropical fruit | |
| Pineapple | 1 cup chunks or diced |
| Papaya | 1 cup chunks or diced |
| Bananas | 1 medium (8-9” long) |
| Melons | |
| Cantaloupe | 1 cup diced or melon balls, 1 small wedge (1” thick) |
| Watermelon | 1 cup diced or melon balls, 1 small wedge (1” thick) |
| Honeydew | 1 cup diced or melon balls, 1 small wedge (1” thick) |
| Pome fruit | |
| Apples | ½ large apple, 1 cup sliced |
| Pears | 1 medium pear, 1 cup sliced |
| Quince | 1 medium |
| Fruit “vegetabl[illegible] | |
| Tomatoes | 1 cup raw diced or sliced |
| Cucumbers | 1 cup raw diced or sliced |
| Eggplant | 1 cup cooked |

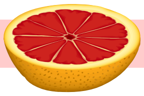
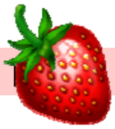
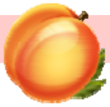
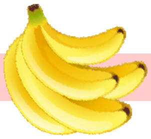
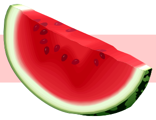
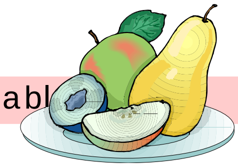
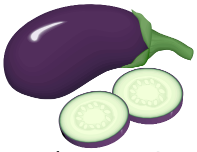

Name:________________________________Date:__________________

# Fruit

| # | Question | Answer |
|---|---|---|
| 1 | Name 2 citrus fruits | Tangerine, tangelo, Clementine, kumquat, orange, grapefruit |
| 2 | Name 3 fruit vegetables | Cucumbers, tomatoes, eggplant |
| 3 | Name 2 types of berries | Strawberry, raspberry, blueberry, blackberry |
| 4 | Name a fuzzy fruit | Peach, kiwi |
| 5 | Name a fruit that grows on a vine | Watermelon or cantaloupe |
| 6 | Name a fruit that grows on a tree in a warm part of the world | Coconut, papaya, mango, guava, bananas, pineapple |
| 7 | True or false: frozen fruit is not as good for you as fresh fruit | False |
| 8 | Name a type of melon | Cantaloupe, honeydew, watermelon |
| 9 | Name a type of stone fruit (with a pit) | Peaches, nectarines, plums |
| 10 | Name a fruit that contains a lot of vitamin C | Orange, grapefruit, tangerine, lemons, strawberries, |


Adapted from: Eat Well and Keep Moving
Copyright © 2001 by Lilian W.Y. Cheung, Steven L. Gortmaker, and Hank Dart

# UMass Boston: GoKids
# Lesson 4 – Food Guide Pyramid – Dairy

Teacher: TBA
Target Audience: Children in grade 3-5 enrolled in GoKids
Date: TBA
Time: 30 minutes

## Timeline:

- Welcome – 1-2 minutes
- Education – 15 minutes
    - What foods are part of the dairy group? Use FGP to explain recommendations and serving sizes for dairy
    - What do dairy foods taste like?
    - What is a serving and how many servings do I need each day?
- Activity – 10-15 minutes

## Materials needed:

- Food sample: calcium rich foods from the dairy group
- Plates, cups, napkins, utensils, snack
- Activity 4: Dairy
- Handout 4: Dairy

## Food models needed:

- 2D and 3D food models

## Welcome Message:

Hello. **QUESTION: How are you doing this week?**

Before we start our lesson for today, I would like to have everyone in the class stand up and stretch!
Ask students to stand up and follow a series of guided stretches (upper body stretches and taking deep breaths). Now I would like to have everyone in the class go around and tell us about a new food that you ate last week. **QUESTION: What did you like or not like about this food?**

## Education:

Today, we are going to talk about dairy foods.

---

**QUESTION: Which foods are considered dairy foods?** (3)
Examples: milk, yogurt, cheese, milk-based desserts

We need dairy foods in our diet in order to get some important vitamins, minerals, and nutrients.
**QUESTION: Can someone name those vitamins, minerals, and nutrients?**

- Dairy foods are naturally high in calcium.

**QUESTION: Why do we need calcium?** Calcium helps our bodies to build strong bones and teeth

- Dairy foods have vitamin D.

**QUESTION: Why do we need vitamin D? ________________________________-**

- Dairy foods have protein and carbohydrates.

**QUESTION: Why do we need protein?** Protein helps to keep us feeling full and carbohydrates gives us instant energy.

---

Let's take a look at this handout on dairy and serving sizes, then we will sample a dairy food. Let's take a look to see how many servings per day we need of dairy foods and what counts as a serving. In general, children your age need _____servings of dairy each day. (Use the 2D and 3D food models)

### Milk

There are many different types of milk that you will find when you go to the grocery store.
**QUESTION: Can someone name some different types of milk?**
Examples: skim milk (fat free), 1% milk (low-fat), 2% milk (reduced fat), whole milk, Lactaid milk, soy milk, chocolate milk, strawberry milk, rice milk, almond milk

**QUESTION: Can someone tell us which types of milk are the healthiest?**
Examples: low fat milk that has does not have added sugar

### Yogurt

There are many different types of yogurt that you will find when you go to the grocery store.
**QUESTION: Can someone name some different types of yogurt?**
Examples: fruit flavored yogurt, yougurt with candy pieces or granola, Greek yogurt, soy yogurt, low-fat yogurt, fat free yogurt, high fat (regular) yogurt

**QUESTION: Can someone tell us which types of yogurt are the healthiest?**
Examples: plain low fat or fat free yogurt is the healthiest because it is low in fat and sugar. Greek yogurt is another good choice because is is high in protein to keep you feeling full.

### Cheese

There thousands and thousands of different types of cheese that you will find when you go to the grocery store.
**QUESTION: Can someone name some different types of cheese?**
Examples: Ricotta cheese, cream cheese, Swiss cheese, American cheese, Cheddar cheese, Provolone cheese, cottage cheese, cheese sticks, etc.

**QUESTION: Can someone tell us which types of cheese are the healthiest?**
Examples: low fat and fat free cheeses are the best choices because they are lower in saturated fat and cholesterol

Milk-based dairy desserts also contain some vitamin, minerals, and nutrients, but they are usually high in sugar and fat. That means that you should limit your intake of these foods and choose other dairy foods like milk, yogurt, and cheese most often.

**QUESTION: What are some ideas for how to include healthful low-fat dairy into meals and snacks?**

- Cheese stick for snack
- Add a slice of cheese to a turkey sandwich
- Have a cup of low-fat yogurt with berries for a snack
- Drink plain low fat milk with meals (up to 3 cups per day)
- Have some cottage cheese and crackers for a snack
- Add some low-fat ricotta cheese to pasta and vegetables

## Activity:

Now I would like to take a few minutes to do a fun nutrition activity!

Distribute the activity to all students. Do the activity as a class. Have the students write their answers in the space provided once you have read the questions aloud.

As an alternative activity, you may choose to play “hangman” with the class using types of dairy foods as the guessing words.

## Questions:

**QUESTION: What questions can I answer for you about today’s lesson?**

## Resources:

(1) http://www.mypyramid.gov/pyramid/index.html

(2) http://aappolicy.aappublications.org/cgi/content/full/pediatrics%3B107/5/1210

(3) http://kidshealth.org/kid/stay_healthy/food/carb.html

# Dairy

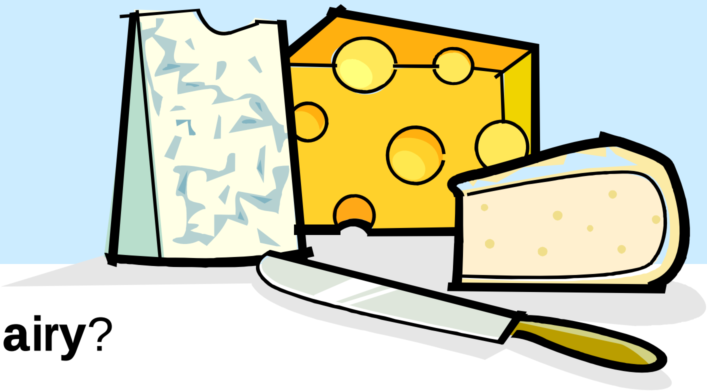

## What counts as one serving of **dairy**?

| Dairy | Serving = 1 cup |
|---|---|
| Milk | |
| Choose skim (fat-free) or 1% (low-fat) milk most often | 8 oz<br>1 half pint carton<br>½ cup of evaporated milk |
| Yogurt | |
| Choose fat-free or low-fat most often | 8 oz container<br>1 cup |
| Cheese | |
| Choose reduced-fat or low-fat cheeses most often | 1/3 cup of shredded cheese<br>½ cup ricotta cheese<br>2 cups of cottage cheese<br>1.5 oz hard cheese, cheddar, parmesa[illegible]ss, mozzarella |
| Milk based desserts – LIMIT | |
| Choose reduced-fat or low-fat types most often | 1 cup pudding made with milk<br>1 cup frozen yogurt<br>1 ½ cups ice cream |

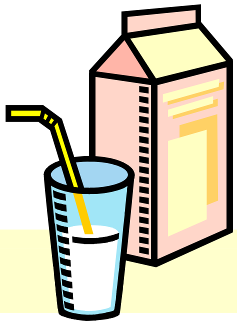

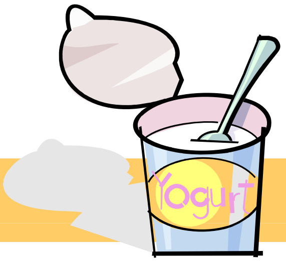


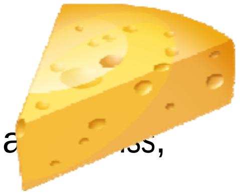

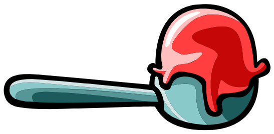

Name:______________________________ Date:______________________

| Dairy | | |
|---|---|---|
| 1 | How many servings of dairy do kids your age need each day? | A. 1 cup<br>B. 3 cups<br>C. 6 cups |
| 2 | Which of the following is the healthiest milk choice? | A. low-fat chocolate milk<br>B. plain white whole milk<br>C. low fat white milk |
| 3 | True or false: You should not eat cheese because it has too much fat? | False |
| 4 | Which of the following is the healthiest type of yogurt? | A. Regular blueberry yogurt with granola<br>B. Low fat yogurt with candy sprinkles<br>C. low fat Greek yogurt with raspberries |
| 5 | True or false: frozen yogurt is healthier than regular yogurt? | False |
| 6 | Which of the following is the healthiest type of cheese? | A. Low fat string cheese<br>B. Cream cheese<br>C. |

Go Kids Week 4 Dairy
Adapted from: Eat Well and Keep Moving
Copyright © 2001 by Lilian W.Y. Cheung, Steven L. Gortmaker, and Hank Dart

# UMass Boston: GoKids
# Lesson 5 – Food Guide Pyramid – Meat & Beans

Teacher: TBA
Target Audience: Children in grade 3-5 enrolled in GoKids
Date: TBA
Time: 30 minutes

## Timeline:

- Welcome – 1-2 minutes
- Education – 15 minutes
    - What foods are part of the meat & beans group? Use FGP to explain recommendations and serving sizes for meat & beans
    - What do meat & beans taste like?
    - What is a serving and how many servings do I need each day?
- Activity – 10-15 minutes

## Materials needed:

- Food sample: calcium rich foods from the meat & beans group
- Plates, cups, napkins, utensils, snack
- Activity 5: Meat & Beans
- Handout 5: Meat & Beans

## Food models needed:

- 2D and 3D food models

## Welcome Message:

Hello. **QUESTION: How are you doing this week?**

Before we start our lesson for today, I would like to have everyone in the class stand up and stretch! Ask students to stand up and follow a series of guided stretches (upper body stretches and taking deep breaths). Now I would like to have everyone in the class go around and tell us about a new food that you ate last week. **QUESTION: What did you like or not like about this food?**

## Education:

Today, we are going to talk about the category of meat & beans on the Food Guide Pyramid.

---

**QUESTION: Which foods are part of the meat & beans group?** (3)
Examples: steak, chicken, fish, eggs, black beans, tofu, nuts, peanut butter

We need protein foods in our diet in order to get some important vitamins, minerals, and nutrients.
**QUESTION: Can someone name those vitamins, minerals, and nutrients?**

- Meat and beans have protein.

**QUESTION: Why do we need protein?** Protein helps to keep us feeling full and it repairs our muscles after exercise

- Meat and beans have iron.

**QUESTION: Why do we need iron?** The body needs iron to transport oxygen from your lungs to the rest of your body. Your entire body needs oxygen to stay healthy and alive. Iron helps because it's important in the formation of hemoglobin (say: hee-muh-glo-bun), which is the part of your red blood cells that carries oxygen throughout the body.

**QUESTION: Which foods are rich in iron?** meat, especially red meat, such as beef, tuna and salmon, eggs, beans, baked potato with skins, dried fruits, like raisins, leafy green vegetables, such as broccoli, whole and enriched grains, like wheat or oats

---

Let's take a look at this handout on meat & beans and serving sizes, then we will sample a food from the meat & beans category. Let's take a look to see how many servings per day we need of meat and beans and what counts as a serving. In general, children your age need _____servings of meat & beans each day. (Use the 2D and 3D food models). We meaure serving sizes using ounces which is a measurement of weight.

### Meats

There are many different types of meat (red) that you will find when you go to the grocery store.
**QUESTION: Can someone name some different types of meat (red)?**
Examples: steak, hamburger, hot dogs, pork, ham

**QUESTION: Can someone tell us which types of meat (red) are the healthiest?**
Examples: lean ground beef (93% lean), lean ham, pork loin

### Poultry

There are many different types of poultry that you will find when you go to the grocery store.
**QUESTION: Can someone name some different types of poultry?**
Examples: chicken, turkey, duck

**QUESTION: Can someone tell us which types of poultry are the healthiest?**
Examples: lean meat without the skin. Grilled, baked, roasted.

### Fish & Seafood

There are so many different types of fish and seafood that you will find when you go to the grocery store.
**QUESTION: Can someone name some different types of fish and seafood?**
Examples: salmon, Cod, shrimp, haddock, lobster, tuna,

**QUESTION: Can someone tell us which types of fish and seafood are the healthiest?**

Examples: most fish and seafood are healthy it is the way that it is prepared that can make it less healthy

## Eggs

There are a few different types eggs and egg substitutes. Eggs have two main parts, the white part and the yellow part. The white part has the protein and the yellow part has the vitamins, minerals, and fat. In general both eggs and egg substitutes are healthy for you in moderation. Let's look at some serving sizes.

## Nuts, Seeds, Dry Beans, and Peas

There many different varieties of nuts, seeds, dry beans, and peas that are all very healthy for you.
**QUESTION: Can someone name some different types of nuts, seeds, or beans?**
Examples: black beans, lentils, kidney beans, garbanzo beans, pumpkin seeds, walnuts, peanut butter

**QUESTION: What are some ideas for how to include healthful lean protein (meat & beans) into meals and snacks?**

- Hummus and raw sliced veggies or whole grain crackers for a snack
- Cheese stick with an apple
- Grapes and a yogurt
- Rice cake and peanut butter
- Add beans to a salad

## Activity:

Now I would like to take a few minutes to do a fun nutrition activity!

Distribute the activity to all students. Do the activity as a class. Have the students write their answers in the space provided once you have read the questions aloud.

As an alternative activity, you may choose to play "hangman" or red light/green light with the class using types of protein foods as the guessing words.

## Questions:

**QUESTION: What questions can I answer for you about today's lesson?**

## Resources:


(1) http://www.mypyramid.gov/pyramid/index.html

(2) http://aappolicy.aappublications.org/cgi/content/full/pediatrics%3B107/5/1210

(3) http://kidshealth.org/kid/stay_healthy/food/carb.html

# Meat & Beans

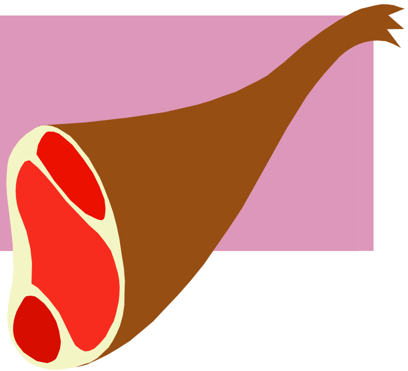

What counts as one serving of **meat & beans**?

| Meat & Beans | Serving = 1 oz |
|---|---|
| **Meats** | |
| Lean beef, pork, or ham | 1 oz cooked lean beef<br>I oz cooked lean pork, ham |
| **Poultry** | |
| Chicken or turkey | 1 oz cooked chicken or turkey<br>1 slice of deli turkey/chicken |
| **Fish & Seafood** | |
| Haddock, shrimp, scallops, salmon, tuna | 1 oz cooked fish or seafood<br>1 can tuna = 3-4 oz |
| **Eggs** | |
| Whole eggs | 1 eggs<br>2 oz egg substitute |
| **Nuts, Seeds, Dry Beans & Peas** | |
| Nuts, nut butters, hummus, soy beans, black beans, pinto beans, tofu | ½ oz nuts (almonds, peanuts, walnuts, pistachios,<br>1 tablespoon of peanut butter<br>¼ cup cooked beans, 2 tablespoons hum[illegible] |

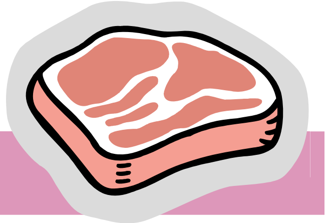

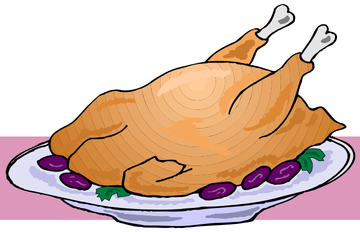

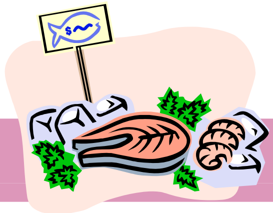

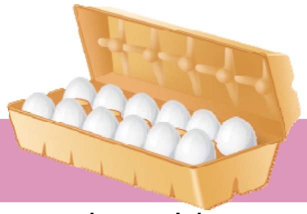

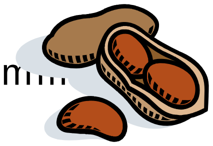

Name:________________________________Date:_______________________

# Meat & Beans

| # | Question | Answer |
|---|---|---|
| 1 | Which of the following is not a source of protein? | A. Eggs<br>B. Fish<br>C. Peaches<br>D. Milk |
| 2 | True or false: vegetables have a lot of protein. | False |
| 3 | Name a type of vegetarian protein. | Beans, tofu |
| 4 | True or false: protein helps to repair muscles. | True |
| 5 | Name a type of animal that produces meat and milk. | Cows |
| 6 | Which would be a healthier choice for deli meat-<br>Turkey or bologna? | Turkey |
| 7 | Which type of protein has more fat than the others? | A. skim milk<br>B. hamburger<br>C. grilled chicken<br>D. low-fat yogurt |
| 8 | Name a type of protein that you could eat for a snack? | ____________________ |
| 9 | Which would be a healthier choice-<br>Baked chicken or fried chicken? | Baked |
| 10 | True or false: you should never eat protein for breakfast. | False |

Go Kids Week 5 Meat & Beans
Adapted from: Eat Well and Keep Moving
Copyright © 2001 by Lilian W.Y. Cheung, Steven L. Gortmaker, and Hank Dart

# UMass Boston: GoKids
# Lesson 6 – Food Guide Pyramid – Oils

Teacher: TBA
Target Audience: Children in grades 3-5 enrolled in GoKids
Date: TBA
Time: 30 minutes

## Timeline:

- Welcome – 1-2 minutes
- Education – 15 minutes
    - What foods are part of the "oils" group? Use FGP to explain recommendations and serving sizes for oils
    - What is the difference between healthful and unhealthful fat? (saturated vs unsaturated)
    - What is a serving and how many servings do I need each day?
- Activity – 10-15 minutes

## Materials needed:

- Food sample: foods from the oils group (i.e. olives, avocado)
- Plates, cups, napkins, utensils, snack
- Activity 6: Oils
- Handout 6: Oils

## Food models needed:

- 2D and 3D food models

## Welcome message:

Hello. **QUESTION: How are you doing this week?**

Before we start our lesson for today, I would like to have everyone in the class stand up and stretch! Ask students to stand up and follow a series of guided stretches (upper body stretches and taking deep breaths). Now I would like to have everyone in the class go around and tell us about a new food that you ate last week. **QUESTION: What did you like or not like about this food?**

## Education:

Today, we are going to talk about fat.

---

**QUESTION: What is fat?** (3)

Fat is one of the three nutrients in food that give us energy.

**QUESTION: Does it surprise you that some fat is good for you?** It might sound weird, but it's true. Fat is an important part of a healthy diet.

- Fat makes food taste good.
- Fat makes a meal more satisfying (which means you may end up eating less).
- Fat fights disease.

---

**QUESTION: Which foods have fat?** (3)

There are 2 main types of fat
-Fat that comes from plants. This is the type of fat that you should choose.
-Fat that comes from animals. This is the type of fat that you should limit.

**QUESTION: Can anyone name some fat that comes from plants?**

Fat that comes from plants includes: (demonstrate with 2D and 3D food models)

- oils, nuts, nut butters, avocados, olives
- liquid at room temperature

**QUESTION: Can anyone name some fat that comes from animals?**

Fat that comes from animals includes: (demonstrate with 2D and 3D food models)

- butter, lard, red meat, fat in milk and cheese
- solid at room temperature

Let's take a look at this handout on fats/oils and serving sizes, then we will sample a few different types of healthful fats. Let's take a look to see how many servings per day we need of healthful fats/oils and what counts as a serving.

**QUESTION: What are some ideas for how to include healthful fat in meals and snacks?**

- Add olive oil and vinegar to green salads.
- Stir-fry vegetables in olive oil.
- Put nuts or seeds on salads and vegetables.
- Add avocados or olives to vegetable-based dishes.
- Nuts and dried fruit for a snack.

## Activity:

Now I would like to take a few minutes to do a fun nutrition activity about fat!

Distribute the activity to all students. Do the activity as a class. Have the students write their answers in the space provided once you have read the questions aloud.

As an alternative activity, you may choose to play “hangman” with the class using types of fat as the guessing words.

## Questions:

**QUESTION: What questions can I answer for you about today’s lesson?**

## Resources:

(1) http://www.mypyramid.gov/pyramid/index.html

(2) http://aappolicy.aappublications.org/cgi/content/full/pediatrics%3B107/5/1210

(3) http://kidshealth.org/kid/stay_healthy/food/fat.html

# Fats/Oils

| OILS/BUTTER/MARGARINE | NUTS ETC. | FATTY FRUITS |
|---|---|---|
| ___Canola | ___Walnuts | ___Avocados |
| ___Olive | ___Pistachios | |
| ___Sesame | ___Cashews | Olives |
| ___Peanut | ___Almonds | ___Black |
| ___Safflower | ___Peanuts | ___Green |
| ___Smart Balance | ___Peanut butter (natural) | ___Greek |
| ___Olivio | ___Cashew butter | |
| ___Margarine | ___Almond butter | |
| ___Butter | ___Soy nut butter | |

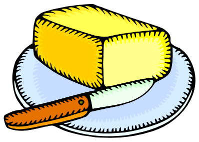
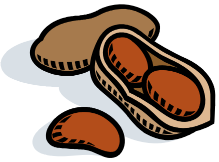
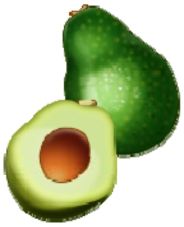
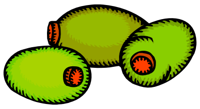

## SPREADS/DIPS/DRESSINGS

___Oil based salad dressing (i.e balsamic vinaigrette)
___Guacamole

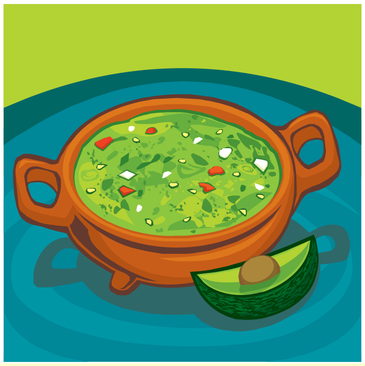
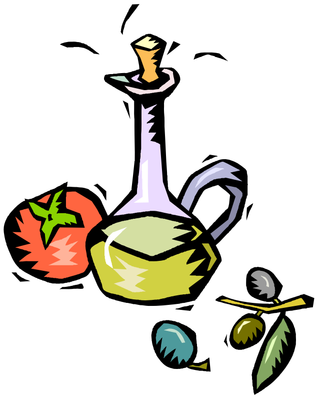
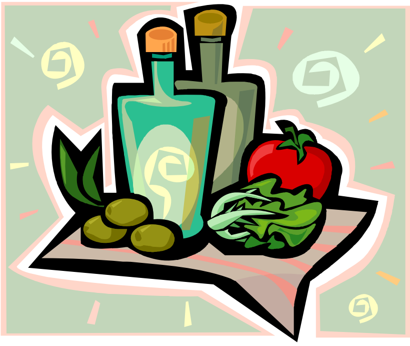

# Fats/Oils

| # | Question | Answer |
| --- | --- | --- |
| 1 | Name a high fat food found in a vending machine. | Cookies or chips |
| 2 | True or false: fats that are liquid at room temperature (such as olive oil) are better for your health than fats that are solid at room temperature (such as butter). | True |
| 3 | Name a high fat liquid that is sometimes served over mashed potatoes. | Gravy or butter |
| 4 | Which type of fat is the least healthy? | A. Olives<br>B. Canola oil<br>C. Avocado<br>D. Butter |
| 5 | Name a high fat dairy food. | Regular cheese or whole milk |
| 6 | Which would be a healthier choice? | A. ice cream<br>B. low-fat frozen yogurt |
| 7 | True or false: the healthiest person is someone who doesn't eat any fat. | False |
| 8 | Name a high fat food that is served in top of pancakes. | Butter |
| 9 | Which lunch is lower in fat? | A. chicken soup and a pear<br>B. pizza and chocolate milk |
| 10 | True or false: fruit is really high in fat. | False |


Adapted from: Eat Well and Keep Moving
Copyright © 2001 by Lilian W.Y. Cheung, Steven L. Gortmaker, and Hank Dart

# UMass Boston: GoKids
# Lesson 7 – Beverages & Snacks

Teacher: TBA
Target Audience: Children in grade 3-5 enrolled in GoKids
Date: TBA
Time: 30 minutes

## Timeline:

- Welcome – 1-2 minutes
- Education – 15 minutes
    - Why is it important to eat snacks?
    - What does a healthful snack look like?
    - Think before you drink. Demonstrate best beverage choices. Use sugar models.
- Activity – 10-15 minutes

## Materials needed:

- Plates, cups, napkins, utensils, snack
- Activity 7: Beverages & Snacks
- Handout 7: Beverages & Snacks

## Food models needed:

- Food models for beverages (bottles with sugar) milk, juice, soda, sports drinks, sugar models

## Welcome Message:

Before we start our lesson for today I would like to have everyone in the class go around and tell us about their favorite snack and beverage.

I will start. My favorite snack is ________________and my favorite beverage is_________________. (go around the room until each student has had a turn to speak).

## Education:

**QUESTION: How many people think that it's important to eat snacks? (Raise your hand if you agree)**

**QUESTION: Why do you think it is important to have small healthful snacks between meals?**

Snacks help to prevent you from becoming so famished that when it is time for your next meal you eat too much and feel stuffed. Your body knows how to process your food more efficiently when you are giving it energy regularly throughout the day.

**QUESTION: What does a healthy snack look like to you?**

(Refer to Snacks handout.) Let's take a look at this handout on healthful snack foods. Using the 2D and 3D food models, demonstrate to the students some examples of healthful snack combinations.

**QUESTION: What do you think about some of these snacks?**

---

**Think Before You Drink: A General Introduction to Beverages**
Many beverages are sweetened with sugar.

**QUESTION: Can you describe why it is not good to eat or drink too much sugar?**

Sugar-sweetened beverages have a ton of sugar! It is also easy to consume a lot of calories from drinks and not even realize it.

Beverage Facts:

- 12 ounces of orange juice has the same amount of calories as eating three chocolate chip cookies!!

**QUESTION: Can you please tell me about some beverages that you see people drinking?**

**QUESTION: What do you think about these choices? Which ones are the healthiest? Why?**

Let's take a look at the Beverages handout. This handout gives examples of how beverage calories can add up during the course of a day.

For each option of the handout, ask the students to identify a healthier alternative to option listed on the left (cover the better choices column on the right).

- Breakfast: A glass of orange juice. **QUESTION: What would be a healthier choice?** An orange
- Coffee Break: A Starbucks Caramel Mocha Coffee with whole milk and whipped cream. **QUESTION: What would be a healthier choice?** Black coffee with half and half and Splenda
- Lunch: Coca Cola. **QUESTION: What would be a healthier choice?** Seltzer water
- Pick Me-Up: Fruit Smoothie. **QUESTION: What would be a healthier choice?** Celery sticks and nut butter
- Gym Break: Vitamin Water. **QUESTION: What would be a healthier choice?** Plain water
- Dinner: Chocolate 2% milk. **QUESTION: What would be a healthier choice?** Plain 2% milk

Let's talk a little bit more about different categories of beverages.

**Soda**
**QUESTION: How much sugar do you think is in one can of regular soda? (ask a volunteer to measure out the number of teaspoons they think are in one can of regular soda)**

There are 10 teaspoons of sugar are in one, 12-ounce can of regular Coca-Cola (ask one volunteer to measure out 10 teaspoons)

**QUESTION: If you like to drink soda, what would be a healthier alternative to regular soda? Diet soda.**

If you enjoy drinking soda occasionally, it is best to choose a diet variety. Sugar-free or low-calorie (≤ 10 calories/serving) beverages such as Crystal Light and Fruit$_2$O are also healthier options.

**Fruit Drinks and Juices**
Many beverages marketed as "fruit drinks" (e.g., Sunny Delight, Kool-Aid, Hawaiian Punch) have 10% or less juice, with sugar as the main ingredient.

**QUESTION: Instead of drinking a glass of juice in the morning what would be a healthier alternative? Eat a piece of fruit.**

It is better to eat your fruit than to drink it. Juice has no fiber. Without fiber, the body does not have to work as hard to digest the fruit juice compared to the whole fruit. The carbohydrate in fruit juice is absorbed very quickly.

**Sports Drinks**
**QUESTION: What have you heard about sports drinks (i.e. Gatorade, Powerade, Vitamin Water, etc.)? Have you ever had any of these drinks?**
Even sports drinks, advertised to enhance performance, have a lot of sugar. Drinking sports drinks has no benefit for most people. Water is usually the best choice!

**Energy Drinks**
**QUESTION: What have you heard about energy drinks (i.e. Red Bull, Sobe, etc.)? Have you ever had any of these drinks?**

There are many "energy drinks", such as Red Bull®, marketed as immediate relief from mental and physical fatigue. However, these "energy drinks" are not only loaded with caffeine but also contain large amounts sugar.

**Coffee and Tea**
Some coffee drinks contain sugar, syrup, or whipped cream. These additions have a lot of sugar and lots of calories.

Let's take a few moments to talk about some popular "coffee shop beverages".
- Black coffee clearly has no sugar. However, lattes, frappacinos, and coolattas have lots of sugar and calories.
- Black, green, or herbal teas are acceptable choices, but beware of the sugar in chai teas.
- Hot chocolate has lots of sugar and should be consumed only as an occasional treat.

Remember, it is important to take control of what is added to your coffee or tea.

**Got Milk?**
Milk is a low glycemic load beverage. There are many different types of milk at the grocery store. There's whole milk, 2% milk, 1% milk, skim or fat free milk, chocolate milk, strawberry milk, soy milk, etc. **QUESTION: How do you know if the milk that you drink is a healthy choice? What type of milk do you drink?** Choose a lower fat milk like 1%.

**Water: The Best Thirst Quencher of Them All!**
Believe it or not your body only needs water to stay hydrated! Water does not cause swings in blood sugar, satisfies thirst, assists with food digestion, and transports nutrients.

Drinking plain water is the best way to satisfy thirst.

## Activity:

Now I would like to take a few minutes to do a fun nutrition activity about beverages!

Distribute the handout to all students. Do the activity as a class. Have the students write their answers in the space provided once you have read the questions aloud.

Discuss the handout previously distributed (beverages list). Ask the students to take the list home and over the next week their assignment is to try a food on the list that they have never had.

## Questions:

**QUESTION: What questions can I answer for you about today's lesson?**

## Resources:


(1) http://www.mypyramid.gov/pyramid/index.html

(2) http://aappolicy.aappublications.org/cgi/content/full/pediatrics%3B107/5/1210

(3) http://kidshealth.org/kid/stay_healthy/food/carb.html

# BEVERAGES

| AVOID<br>Sugar Sweetened Beverages<br>Including: | CHOOSE<br>Non-Sugar Sweetened Beverages<br>Including: |
|---|---|
| • Regular soda | • Water |
| • Gatorade | • Milk, plain (up to 3 cups per day) |
| • Vitamin Water | • Seltzer Water |
| • Powerade | • Fruit Seltzer |
| • Kool Aid | • Fruit20 |
| • Lemonade | • Sugar Free lemonade |
| • Flavored Milk | • Crystal Light |
| • Juice and juice drinks | • Kool Aid Jammers 10 |
| • Iced tea (with sugar) | • Diet Snapple |
| • Iced coffee (with sugar) | • Minute Maid Just 10 |
| • Starbucks iced coffee drinks in bottles | • Propel Fitness Water |
| • Tea (with sugar) | • Diet Iced Tea |
| • Coffee (with sugar) | • Diet Soda (caffeine free) |
| • Milk shakes or large smoothies | • Smart Water |
| • Any drink with more than 10 calories per serving | • Any beverage with 10 calories or less per serving |

# 20 Healthy Snacks

1. Whole wheat English muffin with tomato sauce and shredded low-fat mozzarella cheese
2. Baby grape or cherry tomatoes with 1 tablespoon of ranch dip
3. 1 cheese stick with 1 pear
4. 1 ounce of cashews with 1 cup of green grapes
5. Sliced red and green peppers with 2 tablespoons of hummus
6. 1 serving of soy crisps with 1 ounce of cheddar cheese
7. 1 serving of whole grain crackers with 1 tablespoon of peanut butter
8. 1 cup of cantaloupe with 1 serving of pretzels
9. 1 serving of high fiber cereal with milk
10. 1 container of Greek yogurt with 1 orange
11. 1 Kashi granola bar with 1 cup of raspberries
12. Baby carrots, snow peas, and cucumbers, with 1-2 tablespoons of ranch dip
13. 1 apple with 2 tablespoons of peanut butter
14. 1 hard boiled egg with 1 slice of whole wheat toast with cheese
15. ½ turkey sandwich with baby carrots
16. 5 Triscuits and 1 mini Laughing Cow cheese
17. 2 cups of popcorn with 1 ounce of peanuts
18. 1 cup of cottage cheese and 1 cup of strawberries
19. 1 sugar-free popsicle
20. 10 whole grain tortilla chips with salsa

# Beverages

| # | Question | Answer |
|---|---|---|
| 1 | True or false: you can't gain weight from drinking sugary beverages. | False |
| 2 | How many teaspoons of sugar are in one 12 oz can of regular Coke? | A. 2 teaspoons<br>B. 5 teaspoons<br>C. 10 teaspoons |
| 3 | Name a healthy beverage option that you might find in a vending machine. | Water, diet Snapple, Propel, Fruit 2-0, diet soda |
| 4 | Which has more fiber: | A. a glass of 100% apple juice<br>B. an apple |
| 5 | 4 grams of sugar is equal to how many teaspoons? | 1 teaspoon |
| 6 | Which is the healthiest beverage choice: | A. Propel Fitness Water<br>B. Gatorade<br>C. Powerade |
| 7 | Name the healthiest beverage to choose after exercising. | Water |
| 8 | True or false: 100% fruit juice is just as healthy as eating fresh fruit. | False |
| 9 | Which beverage has the least amount of sugar? | A. Strawberry milk<br>B. Crystal Light<br>C. Orange soda |
| 10 | Name a beverage that is high in fat. | Whole milk |

Go Kids Week 7 Drinks
Adapted from: Eat Well and Keep Moving
Copyright © 2001 by Lilian W.Y. Cheung, Steven L. Gortmaker, and Hank Dart

# UMass Boston: GoKids
# Lesson 8 – Dining Out

Teacher: TBA
Target Audience: Children in grade 3-5 enrolled in GoKids
Date: TBA
Time: 30 minutes

## Timeline:

- Welcome – 1-2 minutes
- Education – 15 minutes
- Activity – 10-15 minutes

## Materials needed:

- Activity 8: Dining Out
- Handout 8: Dining Out

## Food models needed:

- Menu for fast food or chain restaurant

## Welcome Message:

Hello. **QUESTION: How are you doing this week?**

## Education:

Today I would like to talk about dining out.

**Planning Ahead**

The same concepts for choosing foods to eat at home apply to eating away from home. The key is to plan ahead! Let's think about ways to do that.

**QUESTION: How many of you like to go out to eat?**

**QUESTION: Where are some of your favorite places to eat? What do you like to order?**

**QUESTION: If you would like to eat a healthy meal at a restaurant, what would you need to know before going out to eat?**

How the restaurant or eating environment will affect your ability to follow the meal plan. For example, if it is hard for you to resist the fries at McDonald's, don't go there.

**QUESTION: What are some more examples of challenging eating environments?** (Use reflective listening to acknowledge patient challenges.)

- The healthy choices on the menu of your favorite restaurants. Many popular restaurants list their menus on-line, some even have the nutrition information.
- How often you eat out. We recommend eating out no more than 1 time per week. If you eat out more than 1 time per week, ask yourself why you eat out so often.

**QUESTION: What can you do to decrease the frequency of eating out?** (Allow students to respond.)

**Eating at Restaurants**

You can make healthy choices at restaurants if you have some strategies to help you. Be a Detective and Interview Your Server.

**QUESTION: What could you ask your server so that you are able to make healthy choices?**

- What vegetables are included with the meal? Choose a vegetable as a side or order a salad.
- What salad dressings are available? Choose clear dressings that contain healthy oils.
- How is the protein food prepared? It is best to choose a dish that is grilled, baked, broiled, steamed, poached, or rotisserie style. Avoid: fried, crispy, breaded, stuffed, and battered.
- Ask your server if you are unsure how an item is prepared.

**Put It on the Sidelines**.

- If a food is topped with butter, mayonnaise, dressing, sauces, or gravies, ask that the topping be put on the side so that you can decide how much to eat.
- You can make swaps here too. For example, swap creamy dressing for oil and vinegar.

**Slow Down**.

- Listen to your body and stop eating when you are satisfied.

**Beware of Portion Distortion**.

Restaurants often serve huge amounts of food! Don't feel that you have to eat all of the food served.

**Eating at Fast Food Restaurants**

The food available at fast food restaurants is low in quality and taste.  Choose fast food restaurants only if you have absolutely no other options (e.g., when traveling) or as an occasional treat.  You deserve better food!

Let's take a look at the fast food menu. **QUESTION: Which foods look like the healthiest options?**

Good choices at a fast food restaurant are limited and include salads topped with grilled chicken and clear dressings, milk or diet soda, and grilled chicken sandwiches (without the bun or only ½ the bun).  (Listen to ideas from students. Correct misconceptions, as necessary.)

**Eating at School**

The school cafeteria may be a difficult food environment, depending upon the school.

**QUESTION: What are some of the foods offered on your cafeteria menu?  What do you think of these choices?**

Cafeteria meals tend to be high in fat and sugar. Most school lunches lack fresh vegetables, fresh fruit, and lean protein. We strongly recommend making time for breakfast at home and packing lunch for school.

Bringing a lunch to school can be a healthy option because you can decide what to pack.

**QUESTION: What are some tasty lunches you could pack?** (Listen to ideas from students. Correct misconceptions, as necessary.)

- Lettuce topped with chicken, tuna fish and/or cheese and dressed with healthy oil.
- Dinner leftovers.
- ½ Sandwich and soup (thermos).
- Fruit, cheese, and light yogurt.  Add nuts to yogurt for some healthy fat.

## Activity:

Now I would like to take a few minutes to do a fun nutrition activity about dining out!

Distribute the handout to all students. Do the activity as a class. Have the students write their answers in the space provided once you have read the questions aloud.

Discuss the handout previously distributed (fast food menu). Ask the students to take the fast food menu and over the next week their assignment is to come up with an example of a healthy menu item that they could serve at their own restaurant (draw a picture of the meal).

## Questions:

**QUESTION: What questions can I answer for you about today's lesson?**

## Resources:

(1) http://www.mypyramid.gov/pyramid/index.html

(2) http://aappolicy.aappublications.org/cgi/content/full/pediatrics%3B107/5/1210

(3) http://kidshealth.org/kid/stay_healthy/food/carb.html

# The Blue Burger Barn

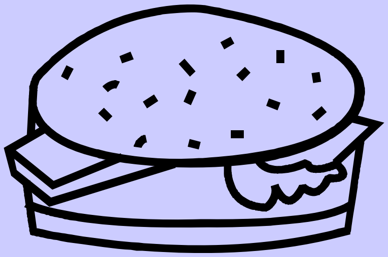

## Menu

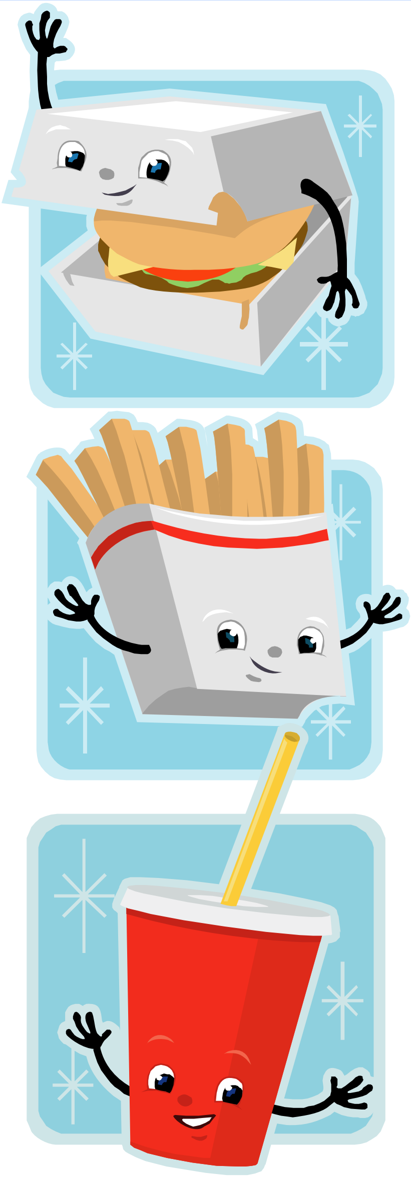

**<u>Burgers</u>**

- Double cheese burger with bacon
- Hamburger
- Cheeseburger

**<u>Sandwiches</u>**

- Crispy chicken sandwich
- Fried fish sandwich
- Grilled chicken sandwich

**<u>Sides</u>**

- Onion rings
- Side salad
- Small order French fries

**<u>Beverages</u>**

- Coke
- Diet coke
- Sprite
- Gingerale
- Fruit punch

# Dining Out

| # | Question | Answer |
|---|---|---|
| 1 | True or false: when you are at a restaurant, it is best to choose water as your beverage choice | True |
| 2 | Which is the least healthy menu option? | A. Chicken nuggets and French fries<br>B. Grilled chicken and corn on the cob<br>C. Pizza and salad |
| 3 | Name a healthy menu item from a fast food restaurant | Salad with grilled chicken (go easy on the dressing!) |
| 4 | True or false: mozzarella sticks are a low-fat appetizer | False |
| 5 | Which is the healthiest menu option? | A. Chocolate chip pancakes<br>B. Whole wheat waffle<br>C. Scrambled eggs with fruit salad |
| 6 | Name a food made with beans that you might find on a menu | Chili, hummus, bean dips, lentil soup, tacos |
| 7 | How many pounds of pasta (like spaghetti and macaroni) does the average America eat each year? | A. 1 pound<br>B. 7 pounds<br>C. 20 pounds |
| 8 | How many teaspoons of fat are there in a McDonald's quarter pounder with cheese? | A. 0 teaspoons<br>B. 3 teaspoons<br>C. 7 teaspoons |
| 9 | How many calories are in a Big Mac and large order of fries? | A. 200 calories<br>B. 750 calories<br>C. 1000 calories |
| 10 | True or false: eating at fast food restaurants most days of the week is a healthy habit. | False |


Adapted from: Eat Well and Keep Moving
Copyright © 2001 by Lilian W.Y. Cheung, Steven L. Gortmaker, and Hank Dart

# UMass Boston: GoKids
# Lesson 9 – Hunger and Fullness

Teacher: TBA
Target Audience: Children in grade 3-5 enrolled in GoKids
Date: TBA
Time: 30 minutes

## Timeline:

- Welcome – 1-2 minutes
- Education – 15 minutes
  - What does it mean to be hungry?
  - What does it mean to be full?
  - Why should I take my time when I eat?
  - What are the five senses that we use when eating?
- Activity – 10-15 minutes

## Materials needed:

- Activity9
- Handout 9
- Food samples for eating exercise

## Food models needed:

- 2D and 3D food models

## Welcome Message:

Hello. **QUESTION: How are you doing this week?**

Before we start our lesson for today, I would like to have everyone in the class stand up and stretch!
Ask students to stand up and follow a series of guided stretches (upper body stretches and taking deep breaths)

## Education:

Today, I would like to talk about hunger and fullness.

**QUESTION: Can someone tell me how you feel when you are really hungry?** (allow time for responses)
You are ready to eat. Stomach is growling. You are able to think about what you would like to eat.

**QUESTION: Can someone tell me how you feel when you are really full?** (allow time for responses)
When you are full you feel satisfied with the meal you ate. You feel comfortable and relaxed.

**QUESTION: What is the difference between being famished and being hungry?** (allow time for responses)
On a scale of 0-10 (0 = empty or famished and 10 = full or stuffed) famished is 0-1 and hunger is 2-4. When you are famished you might feel a little lightheaded because your blood sugar is low, you might get angry easily, you might eat anything in sight because you are so hungry.

**QUESTION: What is the difference between being stuffed and being satisfied?** (allow time for responses)
On a scale of 0-10 (0 = empty or famished and 10 = full or stuffed) stuffed is 9-10 and satisfied is 6-8. When you are stuffed you might feel like your pants are tight, your stomach might hurt a little, and you might feel a little sleepy because you have eaten so much.

It is important to stay somewhere between 2-8. You don't want to become famished because then you might overeat or make poor food choices. You also don't want to get too full or stuffed because when you are stuffed that means that you have eaten much more than your body needs.

It is important to take your time when you eat so that you can digest your food properly and because people who take time to appreciate the food that they eat often end up eating less.

**QUESTION: Does anyone know how long it takes for your body to start to feel full?** (allow time for responses)
When you take time (about 20 minutes) to eat your meal you are able to use all five senses to appreciate and enjoy the food in front of you.

**QUESTION: Does anyone know what our five senses are?** (allow time for responses)

Our five senses are:
1. Seeing
2. Smelling
3. Hearing
4. Touching
5. Tasting

We use all five of these senses when we eat. For example, think about when you getting up in the morning; you want some toast to go you're your breakfast. You put a piece of cinnamon raisin bread into the toaster. You walk away for a minute and then you come back into the kitchen to that sweet smell of cinnamon toasting. The toast pops and you take it out of the toaster, and the toast is that perfectly toasted to a beautiful golden brown. When you take a bite the toast you find it to be sweet and full of flavor. You notice every bite is crunchy and full of flavor until the last bite.

Eating more slowly helps you to know when you are hungry and when you are full. Most of us eat too fast to think about how we use each of our senses when we eat. I would like to do a little activity today using a list of adjectives (**QUESTION: What are adjectives?** Describing words). We will use this list of words to describe the sample food. You can write as many adjectives as you would like to describe the food.

Take about 5-10 minutes to do the activity.

**QUESTION: What kinds of words did you choose to describe the food?** Sight, Smell, Sound, Taste, Texture

## Activity:

Now I would like to take a few minutes to do a fun nutrition activity about hunger and fullness!

Distribute the handout to all students. Do the activity as a class. Have the students write their answers in the space provided once you have read the questions aloud.

Discuss the handout previously distributed (mindful eating adjective list). Ask the students to take the list home and over the next week their assignment is to use the list to descibe one meal or snack that week.

## Questions:

**QUESTION: What questions can I answer for you about today's lesson?**

## Resources:


(1) http://www.mypyramid.gov/pyramid/index.html

(2) http://aappolicy.aappublications.org/cgi/content/full/pediatrics%3B107/5/1210

(3) http://kidshealth.org/kid/stay_healthy/food/carb.html

# Hunger and Fullness

| SENSES | FOOD ADJECTIVES | |
|---|---|---|
| Sight | • What color is it?<br>• What shape is it?<br>• How does it appear? | Red, pink, orange, yellow, beige, tan, brown, green, blue, purple, peach, Round, square, oval, wavy, round, flat, angled, crinkled, broken, clear, bright, dull, glossy, shiny, thick, petite, etc. |
| Smell | • How does it smell? | Clean, burnt, fishy, musty, moldy, nutty, earthy, fresh, sharp, sweet, sour, etc. |
| Sound | • Any sounds? | Clink, crackle, grate, gurgle, hiss, hum, snap, swish, thump, etc. |
| Touch | • What does it feel like when you touch it or chew it? | Fluffy, fizzy, dull, smooth, oily, greasy, hot, cold, cool, warm, dry, creamy, buttery, thick, thin, rubbery, tough, sticky, etc. |
| Taste | • Describe the taste. | Appetizing, appealing, aftertaste, bitter, dry, sharp, sour, spicy, mouthwatering, sweet, tart, sour, tasteless, zesty, etc. |

Directions: Choose a food. Write the name of the food in the box below. Examine the food. Write descriptive words (adjectives) for the sight, smell, sound, touch, and taste.

| SENSES | FOOD:________________________________________ | |
|---|---|---|
| Sight | • What color is it?<br>• What shape is it?<br>• How does it appear? | |
| Smell | • How does it smell? | |
| Sound | • Any sounds? | |
| Touch | • What does it feel like when you touch it or chew it? | |
| Taste | • Describe the taste. | |

# Hunger and Fullness

| # | Question | Answer |
|---|---|---|
| 1 | Describe a chocolate chip cookie. | ______________________________ |
| 2 | Which words describe the way a food looks? (Sense: Sight) | Sour, Small, Rectangle, Crispy, Orange |
| 3 | How long should you take to eat a meal? | A. 5 minutes<br>B. 10 minutes<br>C. 20-30 minutes |
| 4 | Which words describe the way a food smells? (Sense: Smell) | Flowery, Yellow, Brown, Nutty, Citrusy |
| 5 | Why is it important to take your time when eating? | You enjoy your food more and you eat less. |
| 6 | Which words describe the way a food sounds? (Sense: Hearing) | Snap, Soft, Crackle, Pop, Blue, Squish |
| 7 | Describe a kiwi. | _________________________________ |
| 8 | Which words describe a foods flavor? (Sense: Taste) | Green, Sweet, Round, Bitter, Sour, Spicy |
| 9 | Describe a carrot. | _________________________________ |
| 10 | Which words describe a foods texture? (Sense: Touch) | Crunchy, Square, Sticky, Dry, Salty, Clean |


Adapted from: Eat Well and Keep Moving
Copyright © 2001 by Lilian W.Y. Cheung, Steven L. Gortmaker, and Hank Dart

## UMass Boston: GoKids
## Lesson 10 – Goal Setting

Teacher: TBA
Target Audience: Children in grade 3-5 enrolled in GoKids
Date: TBA
Time: 30 minutes

## Timeline:

- Welcome – 1-2 minutes
- Education – 15 minutes
  - o What are goals?
  - o How do I set a S.M.A.R.T goal?
- Activity – 10-15 minutes

## Materials needed:

- Lesson 12 Activity: Goal setting
- Lesson 12 handout: goal setting
- Food samples for goal setting

## Food models needed:

- Food models are not needed for this lesson

## Welcome Message:

Hello. **QUESTION: How are you doing this week?**

Before we start our lesson for today, I would like to have everyone in the class stand up and stretch!
Ask students to stand up and follow a series of guided stretches (upper body stretches and taking deep breaths). Now I would like to have everyone in the class go around and tell us about a new food that you ate last week. **QUESTION: What did you like or not like about this food?**

## Education:

Today I would like to talk to you about setting goals. (4)

**QUESTION: What is a goal?** (allow students time to respond)
You set a Goal whenever you say, "I want to..." Your Goal might be that you want to get a pet dog. Or that you want to learn to dance - or to play football well. Or your goal might be to get better grades at school.

**QUESTION: Why is it important to set goals?** (allow students time to respond)
When you have a Goal, it makes you feel full of energy. Having a Goal makes you feel good about yourself and about everybody else. It makes life much more FUN!

**QUESTION: Can someone please give me an example of a goal that you have set?** (allow students time to respond)

**QUESTION: Why do you think that some people are able to achieve their goals and other people have trouble reaching their goals?** (allow students time to respond)

**QUESTION: What makes something a "good" goal? (allow students time to respond)**

It's always good to have a Goal. But there are ways to make Goal-setting even more fun! Good goals are sometimes called S.M.A.R.T. goals (write this on the board). Here is what makes a S.M.A.R.T. goal smart.

**A S.M.A.R.T. Goal says EXACTLY what you want. It is SPECIFIC**. For instance, you might say, "I want a pet." That's not a very exact Goal, is it?

**QUESTION: How could you make this goal more specific?** If you said, "I want a pet DOG," that would be a S.M.A.R.T Goal. But suppose you said "I want a dog, that will be a little bit smaller than me, it will be brown and black, it will have big pointy ears, and a nice long tail," then that would be an EXCELLENT Goal, because you are saying EXACTLY what you want.

**A S.M.A.R.T. Goal says how much, how many, or how often. It is MEASURABLE.** For instance, you might say, "I want new sports stuff." That's not a very exact Goal, is it?

**QUESTION: How could you make this goal more measurable?** If you said, "I want one new sneakers and one new jump rope," that would be a S.M.A.R.T. Goal because you are saying EXACTLY how many new things you want.

**A S.M.A.R.T. Goal is something that you believe is going to happen. It is ATTAINABLE**. Goals you set that are too far out of your reach, you probably won't stick with. It is best to set smaller goals that you achieve right away so that you can continue to set bigger goals until you reach your ultimate goal. For example, if you said, "I want to lose 20 pounds this week." That is not a safe or attainable goal.

**QUESTION: How could you make this goal more attainable?** A S.M.A.R.T goal would say "I am going to lose 1 pound each week until I reach my goal of 20 pounds."

**A S.M.A.R.T. Goal says says how you're going to get it. It is REALISTIC.** For instance, you might say, "I don't know how to play any sports, but I want to be a professional athlete this year." That is not a realistic goal because you might need more time and practice to become a professional athlete.

**QUESTION: How could you make this goal more realistic?** A S.M.A.R.T. goal would be "I am going to go running every weekend so that I can get better and become a professional runner one day."

**A S.M.A.R.T. Goal says WHEN you want it. It is TIMELY.** If your goal is "I want to get good grades at school," to make it a S.M.A.R.T. Goal, you would say something like: "I want to get grades that are one grade higher than the ones I got in my last report card. And I want to get these grades by the time I get my next report card." That way, you're saying EXACTLY what you want, AND you're saying EXACTLY WHEN you want it!

## Activity:

Now I would like to take a few minutes to do a fun activity on goal setting!

Distribute the handout to all students. Do the activity as a class. Have the students write their answers in the space provided once you have read the questions aloud.

Discuss the handout previously distributed (S.M.A.R.T Goals Diagram). Ask the students to take the list home and talk with parents about setting a healthful goal for themselves and their families.

## Questions:

**QUESTION: What questions can I answer for you about today's lesson?**

## Resources:


(1) http://www.mypyramid.gov/pyramid/index.html

(2) http://aappolicy.aappublications.org/cgi/content/full/pediatrics%3B107/5/1210

(3) http://kidshealth.org/kid/stay_healthy/food/carb.html

(4) http://www.goal-setting-guide.com/smart-goals.html

(5) http://www.googolpower.com/content/articles/goal-setting

# S.M.A.R.T Goals

| | | EXAMPLES | ☺ | ☹ |
|---|---|---|---|---|
| S | Specific | This means that your goal is not too general. It is detailed and exact. | I want to run a mile in 8 minutes. | I want to run fast. |
| M | Measurable | This means that you are able to measure your progress. | I will keep track of how many days I run using a calendar. | I don't need to keep track of how many times I run. |
| A | Attainable | This means that your goal is possible and manageable. | I want to run 5 miles in one hour. | I want to run 5 miles in 2 minutes. |
| R | Realistic | This means that your goal is sensible and practical. It makes sense! | I want to run for 30 minutes on Monday and Wednesday. | I want to run for 3 hours everyday. |
| T | Timely | This means that your goal has a time line. It answers the question, how long? | I want to run 3x/week for 30 minutes each time. | I want to run this week. |

# Goal Setting

| # | Question | Answer |
|---|---|---|
| 1 | When setting a S.M.A.R.T. goal, what does the "S" stand for? | A. Specific<br>B. Special<br>C. Silly |
| 2 | Is this a S.M.A.R.T. goal?<br>I like to eat carrots. | Yes<br>No |
| 3 | When setting a S.M.A.R.T. goal, what does the "M" stand for? | A. Moldy<br>B. Measurable<br>C. Meaningful |
| 4 | Write a S.M.A.R.T. goal for yourself. | ______________________________ |
| 5 | When setting a S.M.A.R.T. goal, what does the "A" stand for? | A. Attainable<br>B. Apple<br>C. Allowed |
| 6 | Is this a S.M.A.R.T. goal?<br>This week I am going to bring a healthy lunch to school three times. | Yes<br>No |
| 7 | When setting a S.M.A.R.T. goal, what does the "R" stand for? | A. Ready<br>B. Race<br>C. Realistic |
| 8 | Is this a S.M.A.R.T. goal?<br>I want to lose weight. | Yes<br>No |
| 9 | When setting a S.M.A.R.T. goal, what does the "T" stand for? | A. Tomato<br>B. Timely<br>C. Terrific |
| 10 | Write a S.M.A.R.T. goal for yourself. | ______________________________ |

Go Kids Week 10 Goal Setting
Adapted from: Eat Well and Keep Moving
Copyright © 2001 by Lilian W.Y. Cheung, Steven L. Gortmaker, and Hank Dart
